# Feasibility of D–D Nuclear Fusion Achieved by Chemical Methods: Quantum Chemical Analysis

**DOI:** 10.1021/acsomega.5c01651

**Published:** 2025-04-17

**Authors:** Siu-Kwong Pang

**Affiliations:** School of Natural Sciences, 4547 University of Lincoln, Brayford Pool, Lincoln LN6 7TS, U.K.

## Abstract

The conceptual design of fusion power plants began decades ago, and significant breakthroughs have been achieved recently. However, the cost of generating energy through controlled nuclear fusion remains extraordinarily high. Cold nuclear fusion achieved by chemical methods offers an alternative approach to cost reduction, but the poor reproducibility of related experiments has led to scepticism about its feasibility. In this study, quantum chemical calculations involving density functional theory (DFT)/basis set (PBE/def2-SVP), geometry optimization, vibrational frequency calculations and relaxed surface scans were performed to calculate Gamow factors and hence estimate D–D nuclear fusion rates in various chemical systems. These systems included free D_2_, D_2_–Pd_44_ clusters, molecular deuterium metal (W, Mo and Cr) complexes, and D_2_–nanocarbon materials (graphene, single-walled carbon nanotubes and fullerenes). A free D_2_ molecule served as a reference point for comparison with other chemical systems. The calculated results indicate that the palladium cluster and metal complexes cannot facilitate the D–D nuclear fusion, whereas carbon nanomaterials can assist with fusing two deuterons together. Remarkably, D_2_ encapsulated within a C_20_ fullerene can exhibit the D–D nuclear fusion rate around 3000 times faster than free D_2_, arising from the compression of the interatomic separation of two deuterium atoms by 11% in a strong and small-sized fullerene cage.

## Graphical abstract

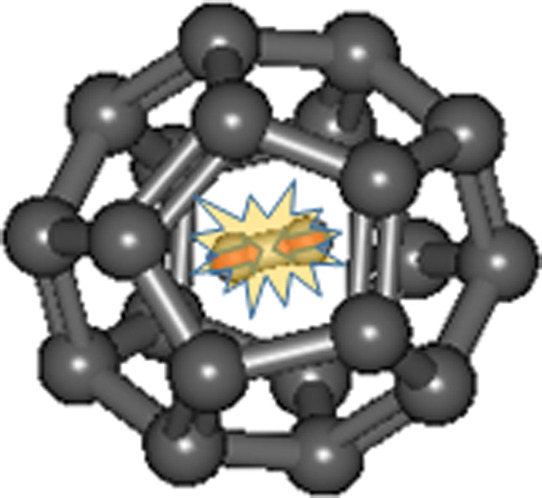

## 1 Introduction

Recently, scientists have made major breakthroughs in green energy technology using nuclear fusion which is the same as the energy process powering stars. The U.S. National Ignition Facility (NIF) announced a milestone in 2022, successfully using 2.05 MJ laser to produce approximately 3.15 MJ of nuclear fusion energy from a deuterium–tritium fuel. In 2024, researchers at Joint European Torus (JET) reported the tokamak device, which generates powerful magnetic field to confine plasma in a toroidal shape, produced 69 MJ of nuclear fusion energy over 5 s using just 0.2 mg of a deuterium-tritium fuel. These breakthroughs made impressive strides in the development of controlled nuclear fusion energy.

The cost of controlled nuclear fusion energy remains exorbitant,^1,2^ primarily due to the handling of extremely high temperatures. Is it possible to achieve nuclear fusion at much lower temperatures than those found in stars? In 1989, an extraordinary claim was made that the electrolysis of heavy water using palladium electrodes produced anomalous heat which was attributed to cold nuclear fusion. But this phenomenon could not be reproduced by most research groups so the claim was dismissed by mainstream researchers. Despite this setback, research on cold nuclear fusion is ongoing, and both successful and unsuccessful cases were reported.^3,4^ Nevertheless, scepticism about cold nuclear fusion persists, largely due to the poor reproducibility of these experiments, leading to a few recent reports on the cold nuclear fusion experiments.^5–7^

Nuclear fusion will occur when two nuclei can overcome their mutual Coulomb barrier through quantum tunnelling to collide with each other. The Gamow factor *G* ^8,9^ (barrier penetration factor) can be regarded as a probability for two nuclei overcoming the Coulomb barrier to undergo nuclear fusion, and it can be expressed as eq 1.

1 $$G=e^{\lambda }$$

The exponent of the Gamow factor is

$$\lambda =-\frac{2}{\hbar }\int_{r_{f}}^{r_{0}}\sqrt{2\mu \left[V\left(r\right)-E\right]}dr$$ 2

where μ is the reduced mass of two nuclei, *r*_0_ is the initial internuclear separation between two nuclei, *r*_f_ is the internuclear separation at which nuclear fusion occurs, *V*(*r*) is the potential energy of a system varying with *r*, and *E* is the energy of a system at *r*_0_. The D–D nuclear fusion rate Λ_D–D_ is mostly governed by the Gamow factor and can be roughly estimated by eq 3.^10^

3 $$∼\Lambda_{D-D}=10^{8}G$$

In the past, certain research groups developed various theoretical models to account for cold nuclear fusion of hydrogen isotopes, and those models are composed of the Gamow factor.^10–19^

This study aims to examine the feasibility of the D–D nuclear fusion achieved by chemical methods through quantum chemical analysis. Various chemical systems were investigated, including free D_2_, D_2_ inside a Pd_44_ cluster, D_2_ on a Pd_44_ cluster surface, D_2_ at a Pd_44_ cluster apex, molecular deuterium tungsten, molybdenum and chromium complexes: W­(CO)_3_(P-iPr_3_)_2_(D_2_), Mo­(CO)_3_(P-iPr_3_)_2_(D_2_) and Cr­(CO)_3_(P-iPr_3_)_2_(D_2_), D_2_ between graphene sheets: C_84_ D_2_ C_84_, D_2_ in single-walled carbon nanotubes: (2,2), (3,3) and (4,4), as well as D_2_ within fullerenes: C_20_, C_40_, and C_60_. The Gamow factors for those chemical systems were calculated using density functional theory (DFT). A free D_2_ molecule served as a reference point for the comparison with other chemical systems. This study can provide more realistic insights into the feasibility of the D–D nuclear fusion achieved by chemical methods compared with previous theoretical studies. Unlike earlier approaches, the Gamow factors were calculated using the data from geometry optimization and relaxed surface scans in this work, whereas previous theoretical studies often lacked geometry optimization for the proposed chemical systems or employed approximate models for potential energy curves. Additionally, vibrational frequency calculations were performed in this study to determine whether the D–D stretching vibrations were present in the geometry-optimized chemical systems or not, as these vibrations can implicate the two deuterons potentially undergo a head-on collision, and hence the D–D nuclear fusion. However, previous theoretical studies did not include this analysis. The videos of H–H stretching vibrations possessed by the geometry-optimized chemical systems are available in Supporting Information. Although the videos show the H–H stretching vibrations, the D–D stretching vibrations can occur in the same chemical systems also but the wavenumbers of D–D stretching vibrations are decreased by a factor of $\sqrt{2}$ since the two hydrogen atoms are replaced by deuterium atoms, doubling the reduced mass. Notably, the H–H stretching observed in the videos involves only the two hydrogen atoms, with all other atoms remaining stationary.

## 2 Computational Methods

All calculations were carried out using the quantum chemical software ORCA 5.0.4.^20^ PBE, which is a popular GGA functional for pure DFT due to its good balance between computational cost and accuracy, was chosen as an exchange–correlation energy functional in restricted DFT calculations.^21–24^ The medium-sized polarized double-ζ basis set def2-SVP, which provides reliable structural predictions and high computational efficiency for large chemical systems, was used for all atoms^25–27^ and the core electrons of heavy elements: Pd, W and Mo were represented by the effective core potential def2-ECP. The geometries of the chemical systems were optimized. The optimized geometries were confirmed when the absence of any imaginary frequencies resulted from vibrational frequency calculations. The xyz coordinates of the optimized geometries are included in Supporting Information. Gabedit 2.5.0 ^28^ was used to look for and view the H–H stretching vibrations generated by vibrational frequency calculations. The *V*(*r*) at different interatomic separations were determined by relaxed surface scans. The scan ranges started from the D–D distances in optimized geometries *r*_0_ to 0.01 Å (10^–12^ m), which is the minimum value allowed by ORCA 5.0.4. If the program can extend to 0.0001 Å, the accuracy of *G* should be further improved. The energy of a system *E* at *r*_0_ was assumed to be the electronic energy of an optimized geometry. The integrals of eq 2 for different chemical systems were evaluated numerically. The figure depicting the Coulomb barriers of the D–D nuclear fusion for the chemical system in this study is available in Supporting Information. The objective of this study is not to study different theoretical models or quantum chemistry methods to find out which one can give highly accurate Gamow factors and hence nuclear fusion rates under ambient conditions. Practically, it is impossible because of a lack of experimental values of the D–D nuclear fusion rates measured under ambient conditions for validation. This work aims to provide completely new insights into the feasibility of the D–D nuclear fusion achieved by chemical methods through a comparative analysis of estimated D–D nuclear fusion rates ∼Λ_D–D_ of different chemical systems with a free D_2_ molecule as a reference point.

## 3 Results and Discussion

### 3.1 Free D_2_ Molecule

The calculated results of D–D interatomic separations *r*_0_, exponents of Gamow factors λ, Gamow factors *G*, and estimated D–D nuclear fusion rates ∼Λ_D–D_ are given in Table 1. From eq 3, the ∼Λ_D–D_ of a free D_2_ molecule can be close to 10^–58^ s^–1^ (8.889 × 10^–59^ s^–1^) while the theoretical models developed in previous studies predicted the rates which can be tens of orders of magnitude different with one another: 10^–47^,^29^ 10^–53^,^30^ 10^–58^,^13^ 10^–64^,^11,31^ 10^–70^,^10,12^ 10^–98^,^18^ 10^–52^/10^–61^/10^–63^/10^–64^,^32^ and 10^–70^/10^–73^/10^–74^/10^–79^.^33^ There are currently no experimental values of the D–D nuclear fusion rates measured under ambient conditions to determine which models are accurate. It is generally understood that the chance of the occurrence of the D–D nuclear fusion under ambient conditions should be extremely low and it cannot be detectable with existing technology. Therefore, cold nuclear fusion can be considered not to occur in a free D_2_ molecule. To explore whether the D–D nuclear fusion can be achieved by chemical methods, this study conducted a comparative analysis of ∼Λ_D–D_ of various chemical systems, using a free D_2_ molecule as a reference point.

**Table 1:**
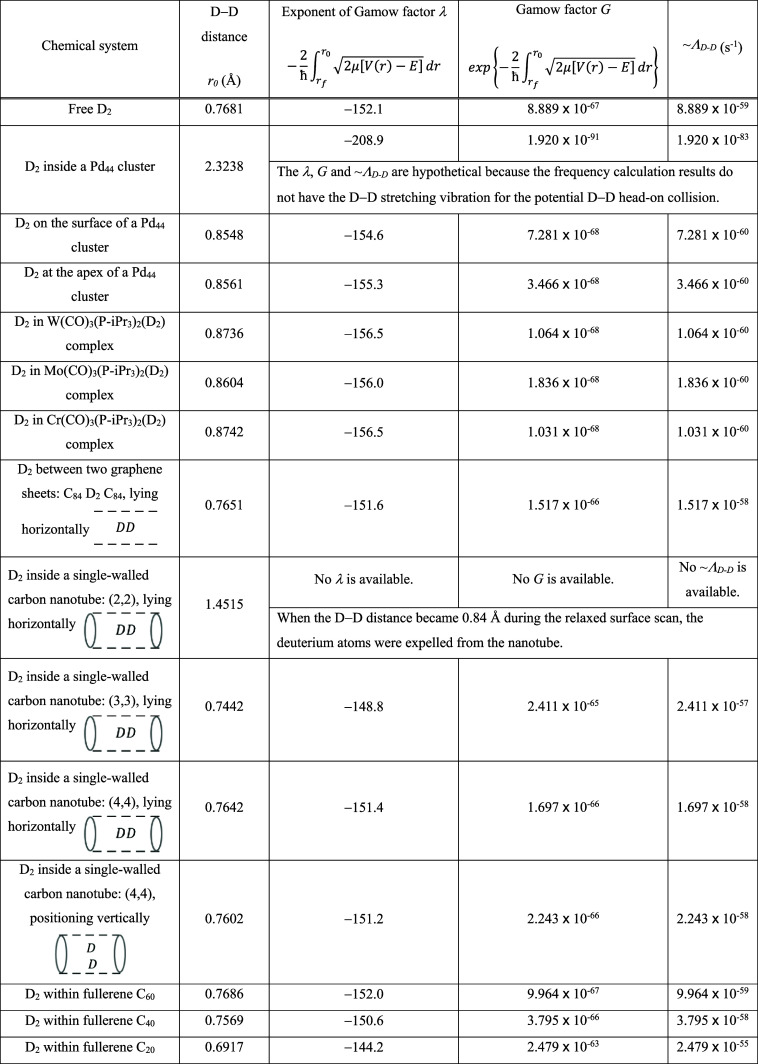
Calculated Values of *r*_0_, λ, *G*, and ∼Λ_D–D_

### 3.2 D_2_ within a Pd_44_ Cluster

The confinement of a D_2_ molecule within a Pd lattice was proposed as a means to increase the D–D nuclear fusion rate under ambient conditions. This idea emerged following the extraordinary 1989 claim regarding the electrolysis of heavy water with palladium electrodes, suggesting that the lattice-caging or lattice-screening by metal lattice electrons could lower the Coulomb barrier for the D–D nuclear fusion. In previous studies, the quantum chemical calculations of a D_2_ molecule in small Pd clusters (Pd_≤6_) deduced that such chemical systems could not achieve cold nuclear fusion because the calculated distances between the two D at small Pd clusters were larger than the D–D distance of a free D_2_ molecule and the two D could not be confined in interstitial sites within the small Pd clusters.^34–38^ Advances in computational power over the past four decades now allow for more detailed investigations. In this study, a D_2_ molecule was placed in the octahedral site at the center of a Pd_44_ cluster initially for geometry optimization. The Pd_44_ cluster ensures the two D can remain inside the palladium lattice during geometry optimization and experience the lattice-caging or lattice-screening generated by the surrounding Pd atoms, unlike the small Pd_6_ cluster. The optimized geometry revealed that the two D were pulled apart, with one remaining in the central octahedral site and the other moving to the adjacent tetrahedral site (Figure 1). The *r*_0_ became 2.3238 Å which is 3 times longer than free D_2_ (0.7681 Å). Additionally, the vibrational frequency calculation for the optimized geometry showed no evidence of the D–D stretching vibration corresponding to the potential D–D head-on collision. Let us assume the D–D head-on collision occurred within a Pd_44_ cluster and the two deuterons finally fused together. Its ∼Λ_D–D_ was calculated to be 1.920 × 10^–83^ s^–1^ which is around 10^24^ times smaller than the nuclear fusion rate of free D_2_ (8.889 × 10^–59^ s^–1^), indicating the lattice-caging or the lattice-screening is not functioning effectively.

**Figure 1:**
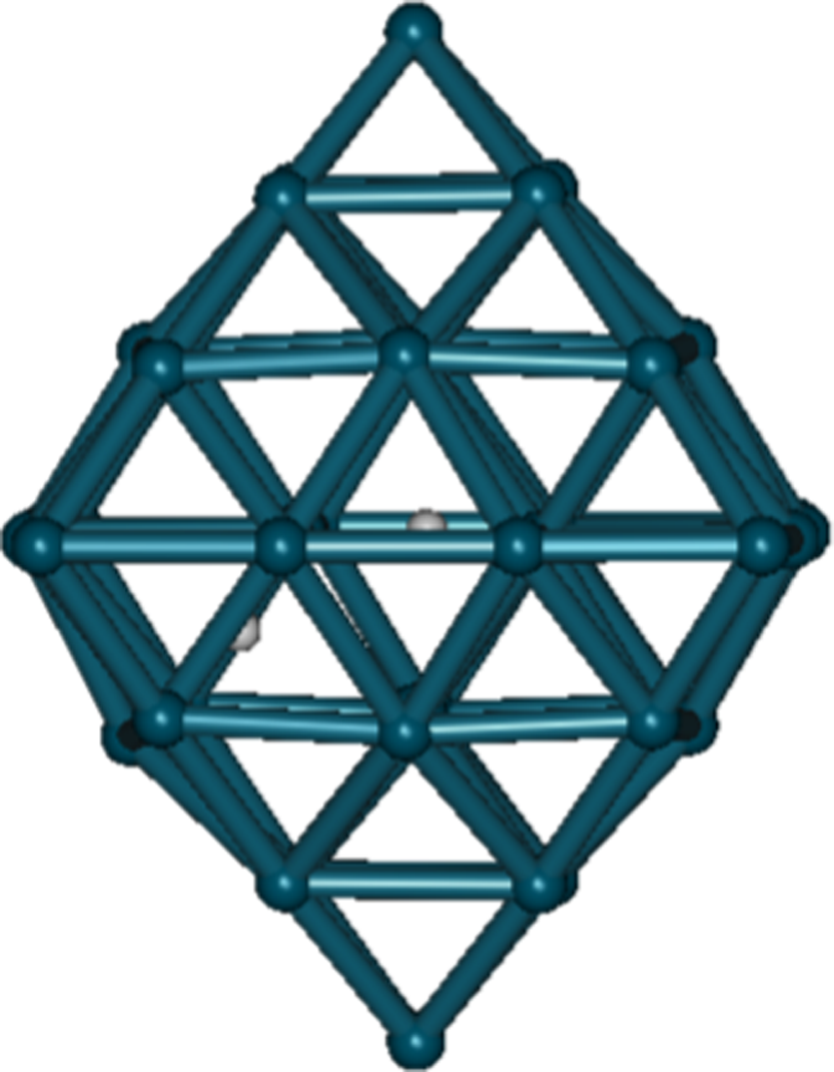
Optimized geometry of D_2_ confined in a Pd_44_ cluster. The two D were pulled apart after geometry optimization.

The system of all octahedral sites inside the Pd_44_ cluster occupied by deuterons was investigated. The initial structure (Supporting Information) for geometry optimization consisted of two deuterons, which were anticipated to undergo the D–D nuclear fusion, in the central octahedral site and other 18 octahedral sites each occupied by a deuteron (total 20 deuterons). After geometry optimization (Figure 2), only three D remained within the Pd_44_ cluster. One was in the central octahedral site, another in an outer octahedral site, and the third in a tetrahedral site adjacent to the central octahedral site. The 17 remaining D were expelled and adsorbed on the surface of the Pd_44_ cluster. The confinement of a D_2_ molecule in an interstitial site of a Pd cluster is not achievable according to the geometry optimization result. The distances between the three D within the Pd_44_ cluster exceed 2.5 Å, and they do not exhibit the D–D stretching vibration for the potential D–D head-on collision. Therefore, the D–D nuclear fusion is unlikely to happen within a Pd lattice.

**Figure 2:**
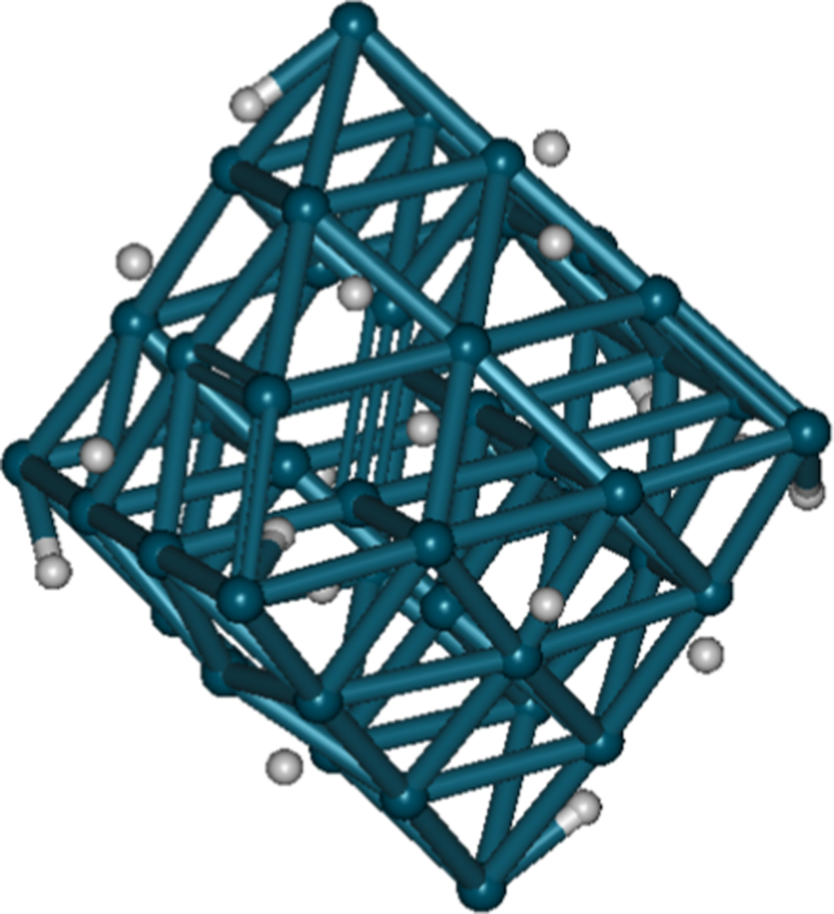
Structure after performing geometry optimization of a system containing two deuterons in the central octahedral site and 18 deuterons in other octahedral sites of a Pd_44_ cluster. The results of the geometry optimization indicate that the initial structure is not preserved: three deuterons occupy octahedral sites, while the remaining 17 are located on the surface of the Pd_44_ cluster.

### 3.3 Intervention of d Orbitals of Transition Metals in D–D Interaction

While the confinement of a D_2_ molecule within a Pd lattice cannot achieve the D–D nuclear fusion, it raises the question: what role does the Pd surface play? Can the d orbitals of Pd assist in the D–D nuclear fusion involving the adsorbed D? To explore this, other two chemical systems containing a Pd_44_ cluster: a D_2_ molecule adsorbed on the surface of a Pd_44_ cluster and a D_2_ molecule adsorbed at the apex of a Pd_44_ cluster were also calculated. Figure 3 displays their optimized geometries. The vibrational frequency calculations confirmed the presence of the D–D stretching vibration implicating the potential D–D head-on collision. Their *r*_0_ (surface: 0.8548 Å; apex: 0.8561 Å) are around 0.1 Å longer than free D_2_ (0.7681 Å). Their ∼Λ_D–D_ (surface: 7.281 × 10^–60^ s^–1^; apex: 3.466 × 10^–60^ s^–1^) are one to 2 orders of magnitude smaller than free D_2_ (8.889 × 10^–59^ s^–1^). Therefore, the intervention of the d orbitals of Pd in the interaction between two adsorbed D on Pd clusters cannot facilitate the fusion of the two deuterons.

**Figure 3:**
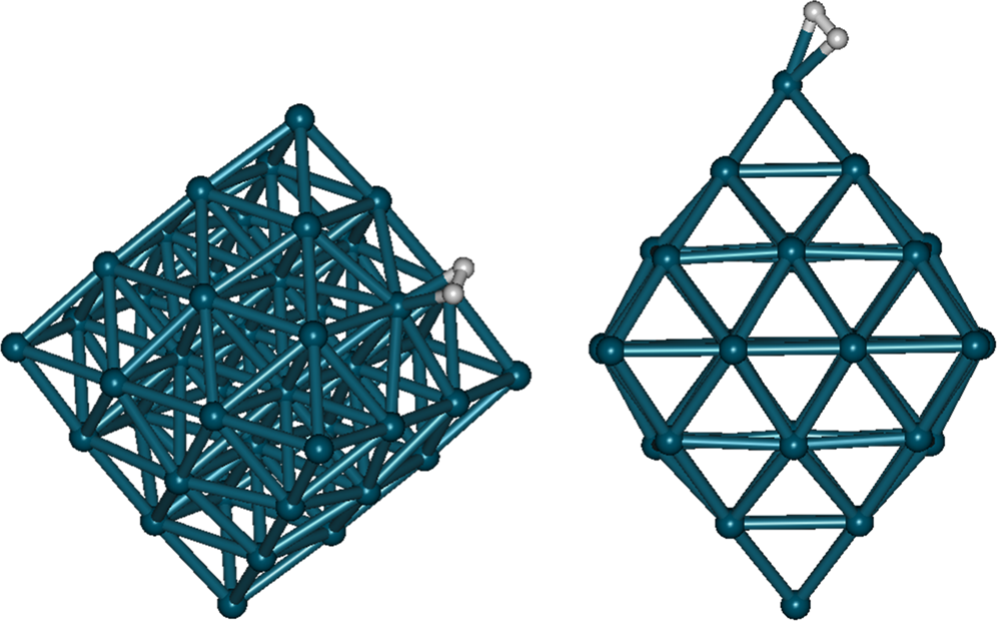
Optimized geometries of D_2_ adsorbed on the surface and at the apex of a Pd_44_ cluster.

Metal complexes containing a molecular H_2_ as a ligand were successfully synthesized four decades ago and the first well-known molecular hydrogen metal complex is W­(CO)_3_(P-iPr_3_)_2_(H_2_)^39^ (Figure 4). As such, three molecular deuterium metal complexes: W­(CO)_3_(P-iPr_3_)_2_(D_2_), Mo­(CO)_3_(P-iPr_3_)_2_(D_2_) and Cr­(CO)_3_(P-iPr_3_)_2_(D_2_) were calculated. They all could exhibit the D–D stretching vibration, potentially leading to the D–D head-on collision. Their *r*_0_ (W: 0.8736 Å; Mo: 0.8604 Å; Cr: 0.8742 Å) are also around 0.1 Å longer than free D_2_. Their ∼Λ_D–D_ (W: 1.064 × 10^–60^ s^–1^; Mo: 1.836 × 10^–60^ s^–1^; Cr: 1.031 × 10^–60^ s^–1^) are one to 2 orders of magnitude smaller than free D_2_. As a result, the d orbitals of Cr, Mo and W (periods 3, 4, and 5 in the periodic table) interacting with the two D in a molecular state cannot enhance the D–D nuclear fusion rate.

**Figure 4:**
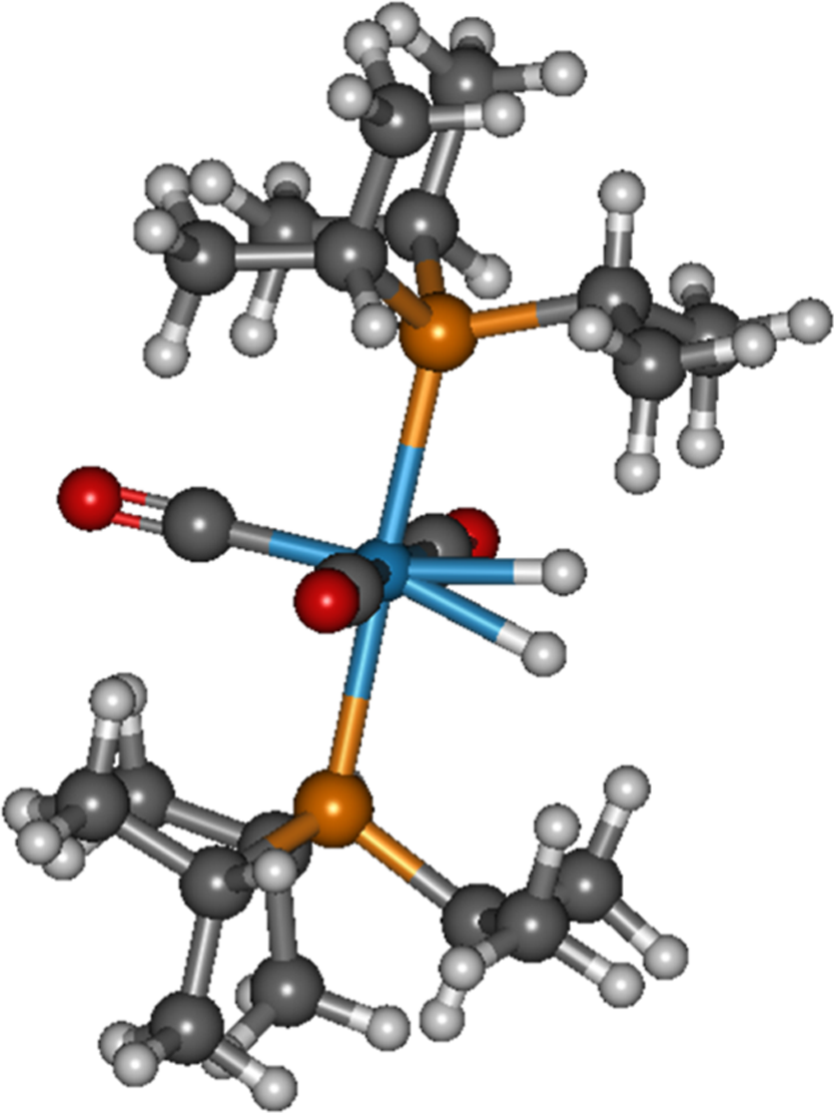
Optimized geometry of W­(CO)_3_(P-iPr_3_)_2_(H_2_).

### 3.4 Carbon-Based Nanoparticles

Three typical carbon-based nanoparticles: fullerenes, carbon nanotubes and graphene have been widely studied in diverse fields of interest for many years. Inspired by the idea of the confinement of a D_2_ molecule within a Pd lattice, D_2_ between graphene sheets: C_84_ D_2_ C_84_, D_2_ inside single-walled carbon nanotubes: (2,2), (3,3) and (4,4), and D_2_ within fullerenes: C_20_, C_40_ and C_60_ were calculated. The results indicate that carbon nanomaterials can increase the D–D nuclear fusion rate, which will be discussed further.

### 3.5 D_2_ between Two Graphene Sheets

The geometry optimization reveals the *r*_0_ of D_2_ lying horizontally between two graphene sheets (0.7651 Å) is slightly (0.003 Å) shorter than free D_2_ (0.7681 Å) (Figure 5). The ∼Λ_D–D_ of D_2_ between graphene sheets (1.517 × 10^–58^ s^–1^) is approximately 2 times faster than free D_2_ (8.889 × 10^–59^ s^–1^). How this “aromatic carbon wrapping” in favor of D–D nuclear fusion is not well understood, awaiting a theoretical explanation for this phenomenon. No optimized structure of D_2_ positioning vertically between two graphene sheets was searched out in the geometry optimization process.

**Figure 5:**
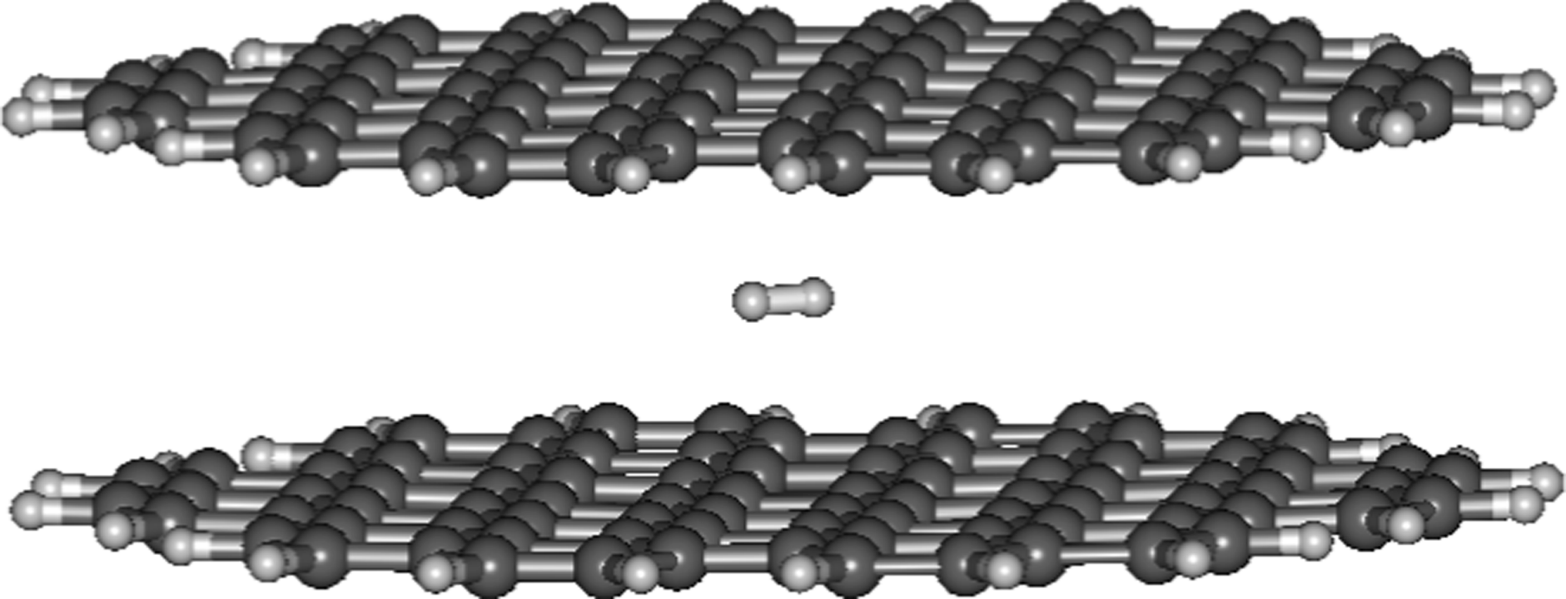
Optimized geometry of D_2_ sandwiched between two C_84_ graphene sheets.

### 3.6 D_2_ inside Single-Walled Carbon Nanotubes

The optimized geometry of D_2_ inside a single-walled carbon nanotube (2,2), which is the thinnest, shows that the two D were pulled apart (Figure 6) with *r*_0_ of 1.4515 Å. When the D–D distance became 0.84 Å during the relaxed surface scan, the two D were expelled from the nanotube (Supporting Information). This can be attributed to their confinement in such a small space, which generated immense pressure that caused the nanotube wall to break, allowing the two D to escape. As a result, no ∼Λ_D–D_ could be calculated.

**Figure 6:**
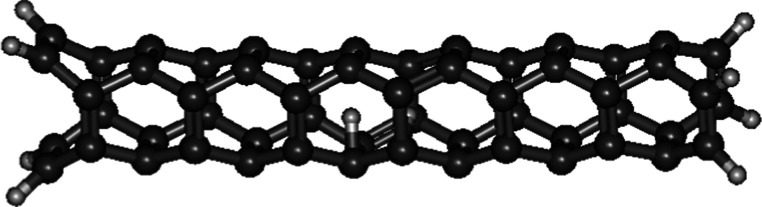
Optimized geometry of D_2_ located inside a single-walled carbon nanotube (2,2). The two D were pulled apart after geometry optimization.

The *r*_0_ of D_2_ lying horizontally inside a single-walled carbon nanotube (3,3) (Figure 7) is 0.7442 Å which is around 0.02 Å shorter than free D_2_ (0.7681 Å). Its ∼Λ_D–D_ (2.411 × 10^–57^ s^–1^) is around 30 times faster than free D_2_ (8.889 × 10^–59^ s^–1^). This thin carbon nanotube can compress the interatomic separation of two deuterium atoms even though they lie horizontally inside the nanotube. This phenomenon is not well understood, awaiting a theoretical explanation.

**Figure 7:**
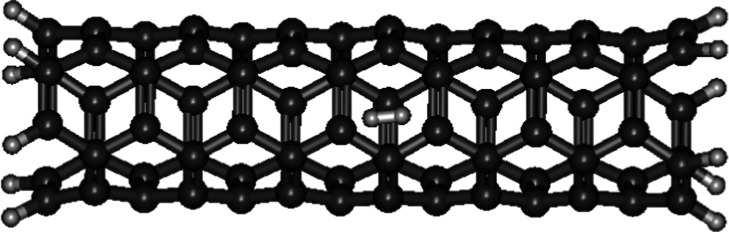
Optimized geometry of D_2_ located inside a single-walled carbon nanotube (3,3).

The *r*_0_ of D_2_ lying horizontally inside a thicker single-walled carbon nanotube (4,4) (0.7642 Å) (Figure 8) is 0.02 Å longer than inside a thinner nanotube (3,3). Its ∼Λ_D–D_ (1.697 × 10^–58^ s^–1^) is 14 times slower than the horizontal D_2_ within a thinner nanotube (3,3). Relative to free D_2_, the *r*_0_ of D_2_ lying horizontally inside a single-walled carbon nanotube (4,4) is slightly (∼0.004 Å) shorter. Its ∼Λ_D–D_ is 2 times larger than free D_2_. The increase in ∼Λ_D–D_ may be attributed to “aromatic carbon wrapping” aforementioned in the scenario of D_2_ sandwiched between two graphene sheets.

**Figure 8:**
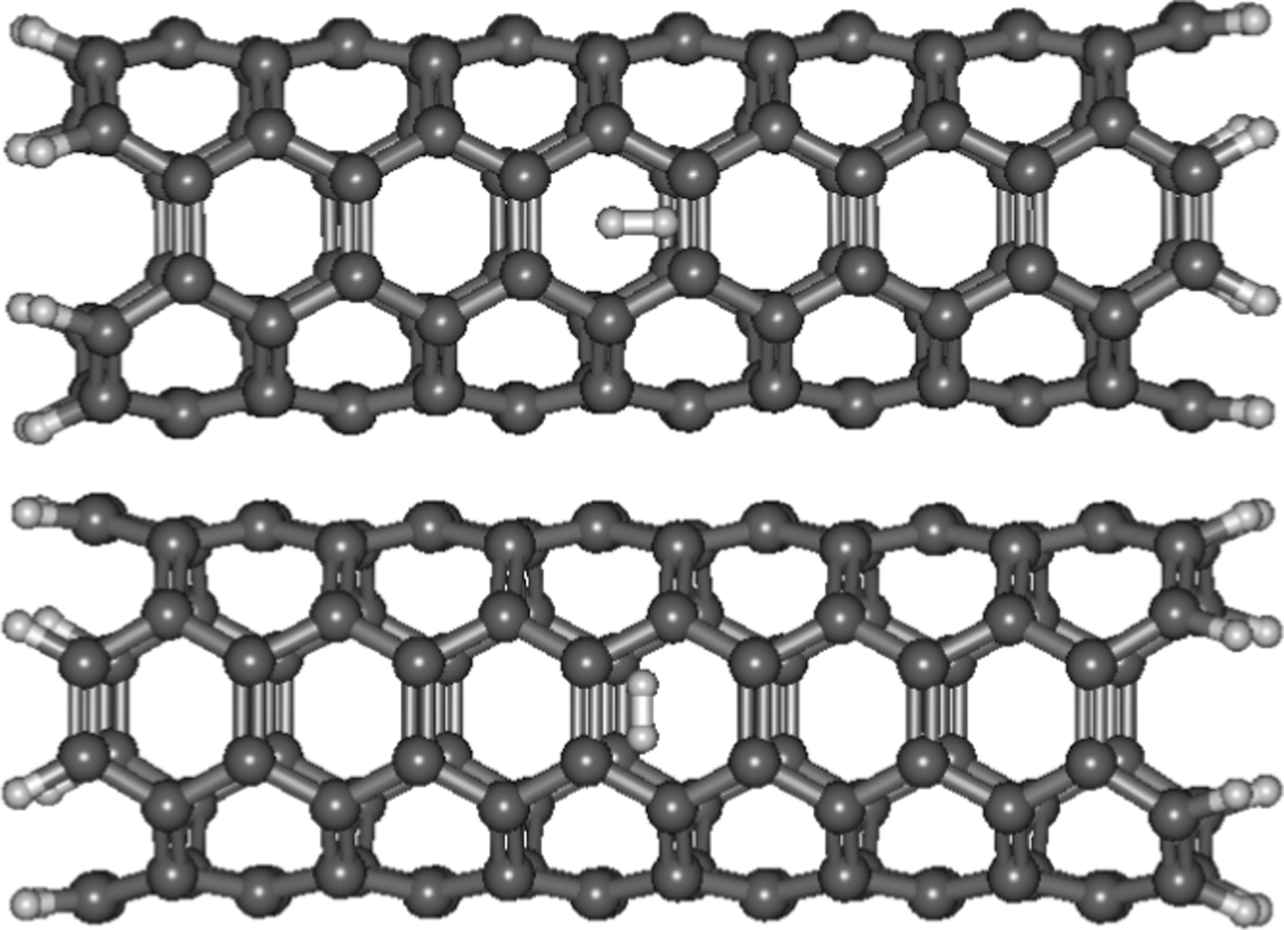
Optimized geometries of D_2_ lying in a single-walled carbon nanotube (4,4) horizontally and vertically.

No optimized structures of D_2_ positioning vertically inside the single-walled carbon nanotubes (2,2) and (3,3) were searched out in the geometry optimization process, except the single-walled carbon nanotube (4,4) (Figure 8). The *r*_0_ of D_2_ positioning vertically inside a single-walled carbon nanotube (4,4) is 0.7602 Å which is 0.004 Å and 0.008 Å shorter than the D_2_ lying horizontally inside the nanotube (4,4) and free D_2_ respectively. Its ∼Λ_D–D_ (2.243 × 10^–58^ s^–1^) is 1.3 and 2.5 times larger than the horizontal D_2_ inside the nanotube (4,4) and free D_2_ respectively. It is suggested that the strong and small-diameter carbon nanotube (4,4) cage can compress the interatomic separation of the two vertically positioned deuterium atoms, bringing them closer together than the horizontal D–D and thereby further increasing the D–D nuclear fusion rate.

### 3.7 D_2_ within Fullerenes

The success in synthesizing a hydrogen molecule trapped within the C_60_ fullerene was reported previously.^40^ From the calculated results in this study, the *r*_0_ of D_2_ within C_60_ (Figure 9) is 0.7686 Å which is slightly longer (0.0005 Å) than free D_2_ (0.7681 Å). Its ∼Λ_D–D_ (9.964 × 10^–59^) is marginally higher than free D_2_ (8.889 × 10^–59^). It is suggested that “aromatic carbon wrapping” facilitates the D–D nuclear fusion, the same as both the D_2_ sandwiched between two graphene sheets and the D_2_ lying horizontally inside a single-walled carbon nanotube (4,4) behaving.

**Figure 9:**
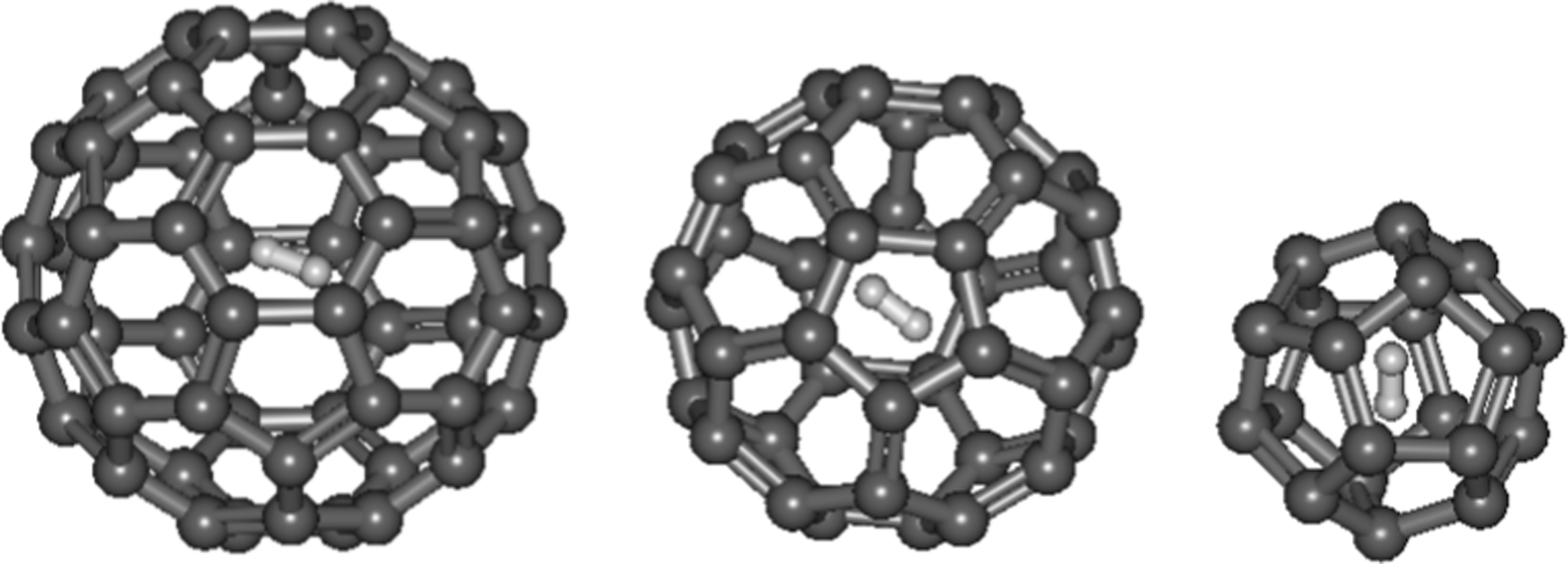
Optimized geometries of D_2_ encapsulated in C_60_, C_40_, and C_20_ fullerenes.

Because of a strong and small-sized fullerene cage, the *r*_0_ of D_2_ within C_40_ (Figure 9) was compressed to 0.7569 Å which is 0.01 Å shorter than free D_2_. Correspondingly, its ∼Λ_D–D_ (3.795 × 10^–58^ s^–1^) is around 4 times larger than free D_2_.

Previously, the smallest possible fullerene C_20_ was proposed to enhance the D–D nuclear fusion rate.^17^ This study found that the *r*_0_ of D_2_ within C_20_ (Figure 9) could be further compressed to 0.6917 Å which is around 0.08 Å shorter than free D_2_ owing to a strong and small-sized fullerene cage. The *r*_0_ is reduced by 11% while its ∼Λ_D–D_ (2.479 × 10^–55^ s^–1^) is around 3000 times faster than free D_2_ (8.889 × 10^–59^ s^–1^). If no molecular cages smaller than C_20_ are capable of encapsulating D_2_, ∼Λ_D–D_ achieved by chemical methods will be limited to being approximately 3000 times faster than that of free D_2_. Can the D–D nuclear fusion be observed from D_2_ within C_20_? The feasibility of the synthesis of D_2_ within C_20_ should be studied. A previous theoretical study reported that neutrons are more efficient than energetic charged particles in transferring kinetic energy to the deuterons in deuterated metals.^41^ Therefore, significant energy generated by the D–D nuclear fusion may be achievable when irradiation of energetic neutrons onto D_2_ within C_20_. Increasing the temperature can excite molecules to higher vibrational energy levels. In this context, the *E* in the exponent of the Gamow factor λ corresponds to these higher vibrational levels, while the *r*_0_ is assumed to be the inner turning points at these higher vibrational energy levels. As a result, the Gamow factor increases, leading to an enhanced nuclear fusion rate. Therefore, it is proposed that D_2_ confined in C_20_ is heated by powerful lasers may induce the D–D nuclear fusion.

## 4 Conclusions

This study investigated the feasibility of the D–D nuclear fusion in various chemical systems using quantum chemical calculations. From the calculated data, the two D within a Pd_44_ cluster were pulled apart which is unfavorable to the D–D nuclear fusion, and the lattice-caging or lattice-screening which was proposed to be capable of facilitating the nuclear fusion of two deuterons exhibited ineffectively in this chemical system. The intervention of the d orbitals of transition metals in the D–D interaction: D_2_ adsorbed on Pd and D_2_ covalently bonded to W, Mo and Cr in metal complexes could not enhance the D–D nuclear fusion rate. Carbon nanomaterials such as graphene, thin single-walled carbon nanotubes (3,3) and (4,4), as well as small fullerenes (number of carbons ≤60) can enhance the D–D nuclear fusion rate. “Aromatic carbon wrapping” was discovered to slightly facilitate the D–D nuclear fusion. Remarkably, D_2_ encapsulated within the C_20_ fullerene can undergo the D–D nuclear fusion at a rate around 3000 times faster than free D_2_ because of the compression of the interatomic separation of two deuterium atoms by 11% in a strong and small-sized fullerene cage.

## Supplementary Material





## Notes

Optimized geometries with their xyz coordinates, initial and optimized geometries of 20 deuterons in a Pd_44_ cluster, geometries of D_2_ before and after expelled from the single-walled carbon nanotube (2,2) during the relaxed surface scan, a plot of *V*(*r*) – *E* against *r* of all chemical systems for illustrating their Coulomb barriers of the D–D nuclear fusion (PDF)
Videos for the H–H stretching vibrations are available in Supporting Information­(ZIP)
