# Supplementary material for: Feasibility of D–D Nuclear Fusion Achieved by Chemical Methods: Quantum Chemical Analysis
Source: ACS Omega. 2025 Apr 17;10(20):20705–12. doi: 10.1021/acsomega.5c01651 (PMC12120576; doi:10.1021/acsomega.5c01651)
Supplement: Supplementary file 1 [file ao5c01651_si_001.pdf]

Feasibility of D–D Nuclear Fusion Achieved by Chemical Methods: Quantum Chemical Analysis

Siu-Kwong Pang

School of Natural Sciences, University of Lincoln, Brayford Pool, Lincoln, LN6 7TS, UK

Supporting Information

xyz coordinates of chemical systems and their optimized geometries

1. xyz coordinates of optimized geometry of D_2

H	0.00000000000000	0.00000000000000	-0.01406012295058
H	0.00000000000000	0.00000000000000	0.75406012295058

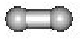

Figure S1. Optimized geometry of D_2.

2. xyz coordinates of optimized geometry of D_2 inside a Pd_{44} cluster

Pd	1.37680029603326	0.04198285991812	1.36338805804430
Pd	4.01572749537545	0.04120206832930	3.83549586067099
Pd	0.06327835690294	-2.10204647260121	2.96435987328288
Pd	1.37693505162357	0.01099911510562	4.07273518608560
Pd	-1.37611998249024	0.01475726143388	1.47281689760903
Pd	-1.40158795129056	0.02139749402940	-1.27675426589562
Pd	1.36390913792649	0.09428273199521	-1.39745594109489
Pd	-0.00171153332899	-1.96469498377250	0.05341803266235
Pd	-0.05314626923437	2.01387246624090	0.07115184351739
Pd	-3.85594036173409	-0.05374721403412	4.09605578307106
Pd	-4.00786373623236	-0.01027276214482	-3.77913832533203
Pd	3.86051989075549	0.13026504016620	-4.02974252684136
Pd	0.06829725989731	-5.58182310050702	0.00002769332967
Pd	-0.09658429283214	5.62237328526651	0.09601747339314
Pd	-2.91586523821312	-2.12538789891013	0.13409988428830
Pd	-0.01872581003130	-2.17053002689832	-3.10484916521552
Pd	2.94417869640117	-2.03047165811429	-0.10367954594791
Pd	2.89843492599834	2.17827143369951	-0.05461661069958
Pd	0.01510214649606	2.11256642674248	3.01687445360013
Pd	-2.99800425752042	2.09602939191337	0.16204500150802
Pd	-0.07051705701109	2.15868944832266	-2.88316933805344
Pd	-4.03773317512339	-0.03800176162682	1.45642011114857

Pd	-1.36247696931932	0.04618637369956	-3.98034187963902
Pd	4.01483006407726	0.10722122252557	-1.40598372847529
Pd	-2.62223556082753	-2.04198808470290	2.80830414220135
Pd	1.44386449269489	-3.81671183934987	-1.46964267039305
Pd	2.66062179593515	2.16194207064986	-2.77946863803213
Pd	-1.46109234996791	3.86250596778651	1.54817666050228
Pd	-2.69904850467165	-1.97004521518542	-2.56251580763044
Pd	1.43953880212976	-3.78213873736248	1.33927120218726
Pd	2.70722096487644	2.02331214361015	2.65775930161952
Pd	-1.47693635268890	3.82066162797283	-1.26265522267814
Pd	2.70085408441615	-1.94664147046401	-2.79975726236351
Pd	-1.37184570834252	-3.91760372860773	1.50459874181288
Pd	-2.69471788150728	1.99356820180340	2.86081697683969
Pd	1.32108904970378	3.90456325582434	-1.38278972858008
Pd	2.74090250448001	-1.93590498727093	2.60119893472409
Pd	-1.35228032137635	-3.79877521466889	-1.30954886430575
Pd	-2.76854384470976	1.98373946395150	-2.54651614173404
Pd	1.34064949154577	3.84130504992260	1.41933946374738
Pd	-1.21320238983750	-0.01607558465169	4.13519170670594
Pd	4.06960336479603	0.07587116026673	1.18157575327054
Pd	1.23378240053675	0.09519318319439	-4.05030209303612
Pd	-4.10249753263494	-0.02648555009490	-1.13135722374769
H	-0.01497214267534	0.11066340988668	0.13116492126258
H	0.23054495099900	-1.32488386328924	-1.67968297738928

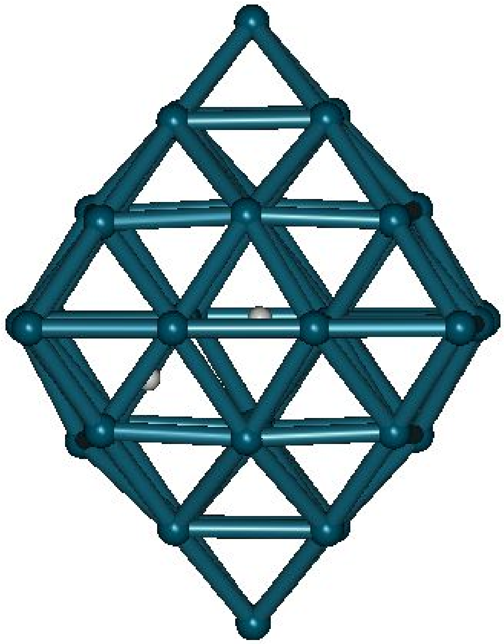

Figure S2. Optimized geometry of D_2 inside a Pd_{44} cluster.

3. xyz coordinates of optimized geometry of D_2 on the surface of a Pd_{44} cluster

Pd	-1.34991320363338	0.77045750407582	1.12095383923878
Pd	-3.94522384206310	2.24308208076799	3.23217339951397
Pd	-0.05326827151368	3.43378440715873	1.13177895454684
Pd	-1.29693123456743	2.38524738234520	3.28966403323649
Pd	1.35021174704030	0.82620579249441	1.07850092532180
Pd	1.35095236133699	-0.76431986997303	-1.12472411535658
Pd	-1.35324242639405	-0.82014404456459	-1.08365927735610
Pd	-0.04772055270555	1.55106722783298	-1.13538475358610
Pd	0.04653681146484	-1.54245545062316	1.12953757729085
Pd	3.94318427931049	2.40207039018790	3.11549810319525
Pd	3.93973398648125	-2.23437785532248	-3.26207488201751
Pd	-3.94213706206368	-2.39318975447539	-3.14511340174464
Pd	-0.14239399828453	4.51694164939285	-3.28934635119490
Pd	0.14033229125378	-4.51531761844722	3.25871761465722
Pd	2.90581402616061	1.75750749189356	-1.29425465240270
Pd	-0.05399618572285	-0.04351380277501	-3.66157532186706
Pd	-3.01303621016143	1.63665671356142	-1.20430585629876
Pd	-2.90474221891310	-1.74982016282991	1.23821658322039
Pd	0.05357690180408	0.05094996449785	3.76326090156638
Pd	3.00722016513219	-1.62926415023256	1.14778822315596
Pd	0.05074442854752	-3.42853164590869	-1.18851384206102
Pd	4.07104770627702	0.85394258405811	0.97504577751696
Pd	1.29107265942651	-2.35617916975634	-3.33128237366594
Pd	-4.07128807736008	-0.83380553957077	-1.00129384925928
Pd	2.63908056168294	3.22521433477404	0.96511551897791
Pd	-1.47741704056597	2.22485583805072	-3.34605370315989
Pd	-2.62928155810923	-3.21960244504095	-0.99257027740485
Pd	1.49626341543494	-2.23353787785838	3.30588203326152
Pd	2.62434610434565	0.06797366623219	-3.37838986683061
Pd	-1.48835664398161	3.85607866746879	-1.10034285553814
Pd	-2.64815823151131	-0.07168871382736	3.34023406202721
Pd	1.47602692229143	-3.85959865125946	1.07266315061478
Pd	-2.72375936607345	-0.04111485523570	-3.29625325994069
Pd	1.29557274797136	3.91569035627959	-1.14217798984171
Pd	2.74526845116926	0.03646358326600	3.25649413513399
Pd	-1.28978923871917	-3.91857592826786	1.11460527630651
Pd	-2.73757946691415	3.11273374801947	1.04525812953001
Pd	1.28610633292933	2.28284737953359	-3.38914844568435
Pd	2.72340792581580	-3.10936042897935	-1.07280137375198
Pd	-1.30419668767388	-2.29001805687008	3.34665596230822
Pd	1.29474741676498	2.43571726075473	3.24705178884406

Pd	-4.07368575730159	0.68806982398639	1.09820710126762
Pd	-1.29510554496635	-2.40970789497231	-3.29215385590883
Pd	4.06972111451913	-0.66952551440293	-1.12435953416576
H	0.46633483039085	-0.13460635206362	5.48522468663210
H	-0.29117636835167	0.26115393662481	5.49891106167260

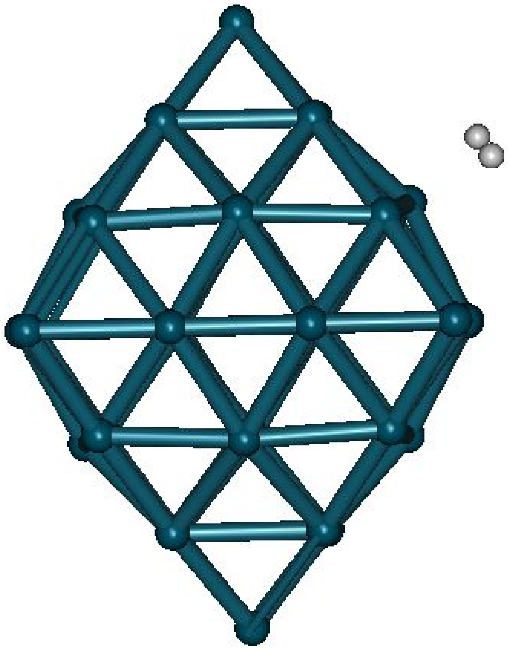

Figure S3. Optimized geometry of D_2 on the surface of a Pd_{44} cluster.

4. xyz coordinates of optimized geometry of D_2 at the apex of a Pd_{44} cluster

Pd	1.91525511811947	0.04866780310723	-0.01337755529048
Pd	5.57110470570331	0.15464016721263	-0.13409885983351
Pd	2.20622872773146	-2.12495988550768	1.98544696905762
Pd	3.86177657898818	0.02185647295961	1.89436555587485
Pd	0.06395412750329	-0.07700813717407	1.95543977859591
Pd	-1.90791142923977	-0.05618936530767	0.10519783174155
Pd	-0.05807945097999	0.08444946050882	-1.88021788110490
Pd	0.05398025323705	-1.91861322724744	-0.04836678348382
Pd	-0.04741659001456	1.92054850502095	0.10395571127246
Pd	0.17296322713249	-0.26162845982960	5.61116610693428
Pd	-5.56633274246105	-0.13905469958674	0.18650276956359
Pd	-0.16540232451017	0.25395688653415	-5.52947075204362
Pd	0.14212032602469	-5.57363847129351	-0.20630017240514
Pd	-0.14079226364972	5.64261215152631	0.33157020113162
Pd	-1.96813947266539	-2.23358724605467	2.10751388568142
Pd	-2.09968707006311	-2.05543400132585	-2.08129427327815
Pd	2.08623886275362	-1.94639926768137	-2.20216794297683

Pd	1.96997257289881	2.23291157352377	-2.01481111150854
Pd	2.08885656191402	2.06049001270079	2.14502090453941
Pd	-2.06508315995301	1.95066862185033	2.26896296580647
Pd	-2.19493135876112	2.12416637582573	-1.89389881638020
Pd	-1.83991290100752	-0.19847331399610	3.87455022002805
Pd	-3.83863508253262	-0.01610586987916	-1.82003597546296
Pd	1.84887283185060	0.21426933854166	-3.81491495724133
Pd	0.16635470115252	-2.13240773614951	3.73775751243035
Pd	0.03995880658442	-3.70610799791117	-2.09336946019055
Pd	-0.16047969755081	2.13015087183863	-3.66912413030196
Pd	-0.03300421435022	3.70427440517341	2.12243105107035
Pd	-3.73080410296194	-2.05783272878909	0.07124093589529
Pd	2.06273503467227	-3.74348493229858	-0.18055241728039
Pd	3.74819356010759	2.05472594209909	0.01473916397391
Pd	-2.07384476637666	3.74873741940633	0.25964449732975
Pd	-0.06138370870409	-1.79968317864054	-3.83913412136097
Pd	0.15784402902718	-3.88511940165264	1.82910583863410
Pd	0.06897852451218	1.80976319628254	3.94019752868296
Pd	-0.15291394066598	3.89457885293482	-1.77383259531314
Pd	3.83574882360653	-1.85792428789415	-0.15014015080004
Pd	-1.86492743958048	-3.84461675291626	-0.06657833628490
Pd	-3.83830060105078	1.85183659006267	0.23593504898351
Pd	1.88560071822635	3.85399246940678	0.14430389423566
Pd	2.07555500518561	-0.09619799065368	3.76153391381539
Pd	3.72850200117947	0.18313791841730	-2.04111352155825
Pd	-2.07366405853321	0.11218329467738	-3.70255868734054
Pd	-3.74116836953338	-0.17818249781360	2.11748145929476
H	-0.30795166399252	6.69308767095546	-1.06728463994268
H	-0.24030068897305	7.24819044903666	-0.41912760319037

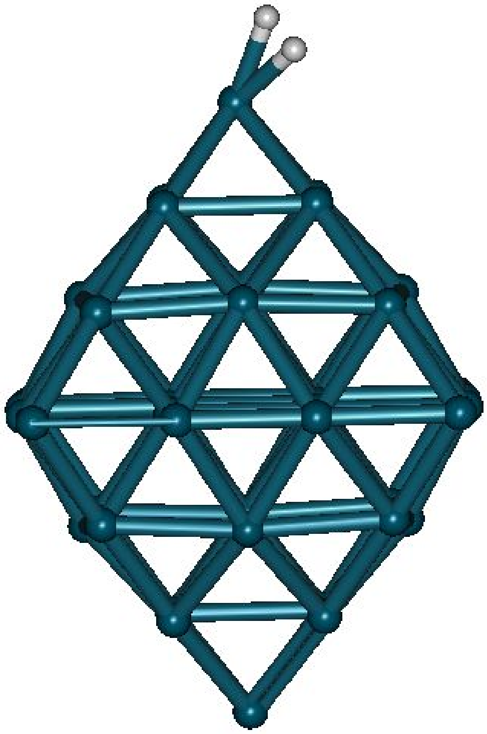

Figure S4. Optimized geometry of D_2 at the apex of a Pd_{44} cluster.

5. xyz coordinates of optimized geometry of D_2 in $W(CO)_3(P\text{-}iPr_3)_2(D_2)$ complex

W	7.26010762931720	3.01453353467543	3.74011335400713
P	8.63467492279899	5.14656782404990	3.90740230407782
P	5.82946555704141	0.91807779019837	3.58330096587398
O	7.57406224188375	3.10067151961523	0.54257274650272
O	6.73345893983364	3.13650346454283	6.90785095810224
O	9.85231766892812	1.23165871570006	4.09176020273694
C	7.46495976652608	3.05811503894798	1.71095356625992
C	6.93575167482246	3.08164380567599	5.75238739996268
C	9.47991070868962	5.40853875749420	5.59365449226951
C	10.20715285257098	6.74310768530865	5.81202858047405
C	10.36571905041444	4.22923146698438	6.01697478829730
C	7.63397984717572	6.75826319809060	3.74791944572859
C	6.96377625484979	6.87566618571539	2.37235174311503
C	6.61369961803981	6.91725227340211	4.88357580368653
C	9.90932719681303	5.37063714695071	2.50856875368699
C	10.94201890935165	4.23720118424971	2.45226795410009
C	10.59489865510329	6.73929441353013	2.38293052325574
C	5.86987336328367	-0.18704038093602	5.13902248501065
C	4.71271273526498	-1.17797126271065	5.34136671623167
C	7.22094665708365	-0.89468063868147	5.31659346217217

C	3.96294188314627	1.23695414375486	3.39627485464609
C	3.41236252938855	2.03298073261486	4.58813608550177
C	3.61774052399076	1.91846186883837	2.06540801516747
C	6.17004724495751	-0.16056769809027	2.05258536005849
C	5.30801890131780	-1.42182868812068	1.89997306947742
C	7.65608238057499	-0.48200491950442	1.84104960339141
C	8.88903744286612	1.89861632771396	3.96203412810952
H	5.49397667924365	3.67561318136658	3.48652450511711
H	5.96706341201852	4.40528762530120	3.56960295940589
H	8.58572033287603	5.39010239709648	6.25633234855462
H	5.79979940488580	0.58678194130966	5.93493951246003
H	3.48696839321249	0.23312907906967	3.40249787678708
H	5.88486092842669	0.55563270273551	1.24984124622746
H	8.28621302691217	0.42404033273384	1.89707026401648
H	5.60703534512496	-2.20878747024652	2.62145136166648
H	3.84230831907275	3.05462798415917	4.62786669403014
H	7.28551017451448	-1.32051243268534	6.34000885095862
H	7.33484860523765	-1.73981561560507	4.60732477842865
H	4.82765951224292	-1.67059903227366	6.33044394235325
H	11.34205249066609	6.90632562047895	3.18325306733340
H	10.46856412390700	3.23961178624102	2.49400146448647
H	7.08904327805022	6.96120797148120	5.88317374666681
H	6.26057779236011	6.03769252729594	2.18558115140498
H	6.38217471665471	7.81963308515996	2.31214727040315
H	7.69378977039889	6.88351948176120	1.53932242059344
H	6.03965203870702	7.85805847983311	4.74868249244579
H	5.88378298485629	6.08135771387327	4.89979787453407
H	11.51379500468287	4.30232365227694	1.50275526403985
H	11.67566675915085	4.30372489317462	3.28085679264737
H	11.14220967694684	6.78674663831335	1.41742074413742
H	9.88834753976032	7.59193880476543	2.39539259650809
H	3.71533055380139	-0.69770623564483	5.33640430364476
H	4.70869783168094	-1.98148292472983	4.57961161376706
H	8.08166061998304	-0.21445685361361	5.18216121889977
H	2.31059568027140	2.13836284393633	4.49880690744836
H	3.62444007566716	1.55295784903642	5.56371725405264
H	8.03996457449319	-1.20782775481064	2.58421734375657
H	7.79724431349161	-0.93126979602468	0.83541544164793
H	4.22380341548920	-1.23387704722549	2.02988753490931
H	5.44680539459911	-1.84829804102068	0.88373860351295
H	9.61235963295984	7.62713557120214	5.50952087493441
H	11.29822721935156	4.17490447110482	5.42042246475930
H	2.53562821749924	2.16546078206283	2.03479724372873

H	3.83286864581685	1.27719561461056	1.18824182706521
H	10.44499315995225	6.86257235125016	6.89040963121123
H	11.17012200886210	6.77713266226689	5.26532248425075
H	10.66153483741554	4.34956313484773	7.08040025566890
H	9.84249849938791	3.26066106635068	5.91908026103022
H	4.17995131276558	2.86646498523971	1.93143376758995
H	8.37541896754605	7.58092541496624	3.83894020852315
H	9.24288957302022	5.24125706856762	1.62652417248730

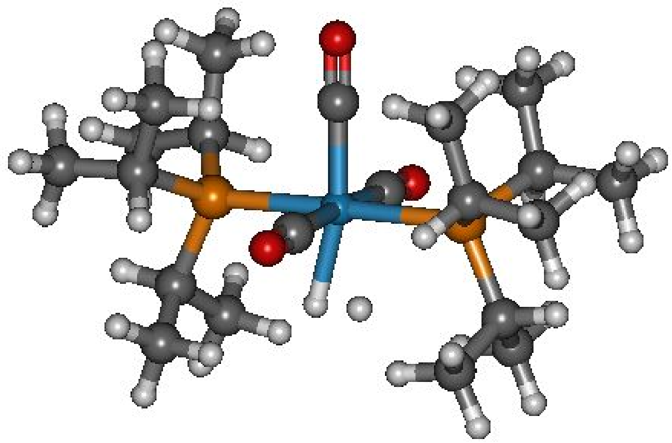

Figure S5. Optimized geometry of D_2 in $W(CO)_3(P\text{-}iPr_3)_2(D_2)$ complex.

6. xyz coordinates of optimized geometry of D_2 in $Mo(CO)_3(P\text{-}iPr_3)_2(D_2)$ complex

Mo	7.27120606412320	2.99687288827351	3.75741018043483
P	8.63000332969924	5.12377443188340	3.90658973471428
P	5.83001120154491	0.92807096784626	3.57999599689078
O	7.54175237025183	3.05835118732042	0.57332852495820
O	6.96083771935803	2.96644417250000	6.93752164742727
O	9.86700171382640	1.24049850942711	4.02279121625920
C	7.43556761518564	3.03875654104229	1.74152044090182
C	7.06795307396449	2.98290675319105	5.76952655000080
C	9.52661893592250	5.38434336766239	5.56719915902942
C	10.23821329453948	6.72897338916522	5.77619716156013
C	10.44652802776320	4.21734922164265	5.95022032023846
C	7.61160741389539	6.72970218002533	3.79275334599944
C	6.89610673872063	6.85459679141931	2.44054531082008
C	6.62796138862474	6.87297997989343	4.96255517621504
C	9.86054421116748	5.37530178506632	2.47264561123988
C	10.89782208049105	4.24907014327572	2.36968246619545
C	10.53786041812998	6.74816162875643	2.34444226638912
C	5.79406290602757	-0.14670043812188	5.15307272176066
C	4.75397591721981	-1.27469672123970	5.22362585579840
C	7.18183919646682	-0.66154007342164	5.55802069279677

C	3.97281681439424	1.26751170777108	3.32828077195419
C	3.35654334881418	2.00498459437446	4.52489865782878
C	3.69920514044705	2.00979768079393	2.01297148091191
C	6.19787172935952	-0.16786441536560	2.06573820283695
C	5.26345659076859	-1.36076718860172	1.81540361312372
C	7.66724847012426	-0.59951290642453	1.97170172618437
C	8.89669087796968	1.89820393591847	3.91057346613498
H	5.50841969676111	3.67244256350215	3.63135204681466
H	5.99072983162625	4.38408840990891	3.66737047749882
H	8.65609752277796	5.34469608447331	6.25967193055606
H	5.50867458600450	0.62662239726445	5.90091392113269
H	3.49325866435684	0.26721545538147	3.26372740285050
H	6.03212187253772	0.57327827652461	1.25208472891474
H	8.35750099405777	0.25915060448143	2.05252879992794
H	5.46034593256587	-2.19229017288435	2.52078577183982
H	3.83707378314403	2.99098656487529	4.69474423917970
H	7.13926595299287	-1.07603985907165	6.58718929631761
H	7.53184523171592	-1.47651621545602	4.89256363015795
H	4.71778854175214	-1.67494547010078	6.25926938887738
H	11.32069187686416	6.89869221900904	3.11353072831366
H	10.43194005670977	3.24754771121856	2.39807399807305
H	7.13653624786770	6.92614201474064	5.94502694105310
H	6.18362478232764	6.02045056840290	2.27249115448718
H	6.31642339415702	7.80094177732106	2.40345934791121
H	7.59891082497140	6.86411267790616	1.58437807143194
H	6.03420103437435	7.80404362411124	4.84686799783278
H	5.91312394072114	6.02537185715962	5.00501595466949
H	11.45046166804973	4.33920068773551	1.41094457979977
H	11.64690900236741	4.30183375634309	3.18524488937666
H	11.04184755381653	6.81559032098312	1.35678482874126
H	9.83293492891451	7.60002500092910	2.40597825521886
H	3.72739089136550	-0.94734534069235	4.96739392814982
H	5.01399944055567	-2.12262008640010	4.56055186290820
H	7.94476773063612	0.13818415374221	5.54885485969721
H	2.27709763805789	2.18948075675982	4.34151444428855
H	3.43813751449278	1.43202122441443	5.46933560777009
H	7.93953453127013	-1.33060016632142	2.75876537495404
H	7.84814525670294	-1.08814951359258	0.99116094071386
H	4.18788953013431	-1.10464976471988	1.88298309275551
H	5.43978008020008	-1.75858311733339	0.79343855285957
H	9.61473050399782	7.60595844748596	5.51314910534190
H	11.36474972412843	4.19037562131922	5.32978696422481
H	2.61064292303888	2.19951220944943	1.90302005649664

H	4.02545443275784	1.43694980777461	1.12287857211338
H	10.51882193240982	6.83634426119913	6.84552722353950
H	11.17646065539605	6.79062124520420	5.19046419544214
H	10.76753452228948	4.32821165359295	7.00734221436977
H	9.94205245071202	3.23913443963974	5.85204543716644
H	4.21406157286215	2.99220836346683	1.98219287751625
H	8.34940634879388	7.55721656158973	3.86581243672136
H	9.16900780996261	5.25471827458836	1.60897157338840

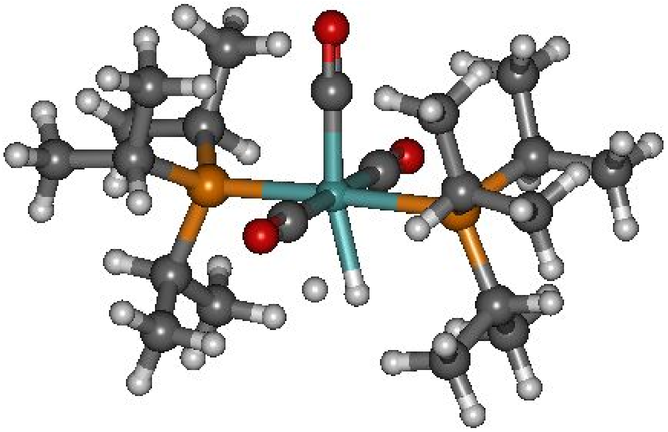

Figure S6. Optimized geometry of D_2 in $Mo(CO)_3(P\text{-}iPr_3)_2(D_2)$ complex.

7. xyz coordinates of optimized geometry of D_2 in $Cr(CO)_3(P\text{-}iPr_3)_2(D_2)$ complex

Cr	7.27130211966949	3.00106515083053	3.74154085852354
P	8.55647244013274	4.98763376104368	3.89354862253350
P	5.92795209056012	1.04964613749303	3.58910239382249
O	7.57824065796085	3.09372381026553	0.71924473767910
O	6.84205496651238	3.00433089305880	6.75001978817843
O	9.74815369657347	1.32368993921017	4.01735675159656
C	7.45327905911479	3.05612401676587	1.88676726074446
C	7.00521470767032	3.01063603681662	5.58656749487837
C	9.37244238794563	5.30402600273063	5.58651274682255
C	10.15615410361941	6.61655919500915	5.72959195304472
C	10.19453703026967	4.12164956744095	6.12038267371851
C	7.55380169910106	6.59531310346623	3.67795234191994
C	6.84175246184316	6.63026625082251	2.31773726329089
C	6.55889038250253	6.82740497911754	4.82292475916393
C	9.89352435381941	5.20756126172080	2.54149778716544
C	11.10458328175191	4.28418109550005	2.74534113377013
C	10.36571981299013	6.63814557889758	2.23312169605011
C	5.99616568791536	-0.11237044303337	5.10741535274627
C	4.80240774086816	-1.05225571397211	5.34161380907567
C	7.31494762868473	-0.89594445913396	5.19840724905704

C	4.05418804148644	1.38557127308282	3.47252113553855
C	3.55389093609474	2.18271789346766	4.68629716526873
C	3.65827227425241	2.07644474872137	2.16106991731718
C	6.19425114431215	-0.00250425965672	2.02374726746745
C	5.29164361045588	-1.23607222764672	1.87189959029042
C	7.66052383606850	-0.36862511113941	1.75184327414890
C	8.77528309159129	1.98306827387862	3.91064528135534
H	5.68019927654991	3.55724774707535	3.47672925458697
H	6.13136646146185	4.27321234237922	3.69607301908076
H	8.46529450945319	5.37272801725311	6.22719591940222
H	6.00215167577213	0.62940707775091	5.93485936676237
H	3.57167749929954	0.38575028593197	3.49249561020309
H	5.90456064848261	0.73620848309579	1.24364510030881
H	8.32935133201112	0.50957611806712	1.78724589132537
H	5.58716876852011	-2.04651311442577	2.56839713225641
H	3.95934371893143	3.21425867542220	4.68908997396973
H	7.40543138752059	-1.34821878175282	6.20856742715622
H	7.34598384484433	-1.73125580283667	4.46932634473350
H	4.95610780215365	-1.59644001528011	6.29799251004079
H	10.90221355632320	7.10133085159877	3.08396338344935
H	10.82464005640308	3.24044174154263	2.97524114527513
H	7.05584622842526	6.96811586001030	5.80277983647356
H	6.04283357524505	5.86397861507854	2.25743739397078
H	6.36171327604088	7.62014159339305	2.16611962125558
H	7.52613731708553	6.45593916584409	1.46426204813851
H	5.95772177294556	7.73965408993140	4.62423046112839
H	5.84838351758893	5.98079895482315	4.92699767414769
H	11.71701113187112	4.26914986892397	1.81903480318298
H	11.76336302904655	4.64980129250100	3.55950327340886
H	11.07956999804635	6.60527631266007	1.38240719539733
H	9.54546231993663	7.31898528952211	1.93554023582247
H	3.83515049123648	-0.52142309193608	5.42873632271085
H	4.70115017105989	-1.81861702001343	4.54872090302765
H	8.20796892766384	-0.26621093327081	5.03455726158712
H	2.44669473434825	2.26286411443493	4.65747205481854
H	3.83090729881797	1.72076929557849	5.65431666345588
H	8.04743713950483	-1.11682521858434	2.47033852428893
H	7.74421354502103	-0.81137105443140	0.73680539058755
H	4.21726386099432	-1.02084295564022	2.03530192609855
H	5.39046004830220	-1.64533268751278	0.84392739797964
H	9.59900045266049	7.50531826672258	5.37343803573401
H	11.13923834084148	3.98090823122551	5.56064333907474
H	2.57487013368904	2.31956272580232	2.17334961627190

H	3.84260631900366	1.44449056872567	1.27031004764608
H	10.39887386142349	6.79014065216512	6.79969419057717
H	11.12141807330011	6.57691852333020	5.18561709022814
H	10.46361212666736	4.31284226466903	7.18071201779333
H	9.63673189482796	3.16909781463460	6.08572437901851
H	4.20775478766824	3.02991702340694	2.01392448939558
H	8.29988052523733	7.41719656333751	3.69932544315014
H	9.34728532000570	4.83623549408850	1.64767997991018

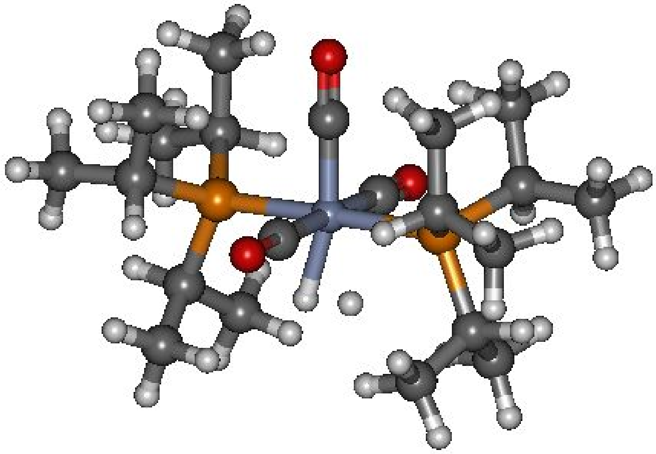

Figure S7. Optimized geometry of D_2 in $Cr(CO)_3(P\text{-}iPr_3)_2(D_2)$ complex.

8. xyz coordinates of optimized geometry of D_2 between two graphene sheets: C_{84} D_2 C_{84}, lying horizontally $\overline{\underline{DD}}$

C	-7.79318477995491	-0.96236280631365	1.58843327725002
C	-7.14309367472484	-0.99051774485558	0.32270199656037
C	-7.88049752363894	-0.97515697161950	-0.89447985182734
C	-5.81107433566404	-1.03524718492030	-2.20565506955743
C	-7.23550817992919	-0.99549539799993	-2.11304407010635
C	-5.13176066003586	-1.04613303278326	-3.48392881575648
C	-5.85182074021678	-1.01795694960487	-4.71690565353340
C	-3.78138208147737	-1.03985066951896	-6.00299884256013
C	-5.20261035407569	-1.01560424630086	-5.93336468968734
C	-3.09604360528851	-1.02729159582900	-7.25019831711236
C	-5.63662447871233	-1.00731559972613	2.75197300339996
C	-7.06423247267781	-0.96994196416633	2.75887637601012
C	-4.95665069556084	-1.04272493781365	1.49371891828777
C	-5.71242870409627	-1.03265389875742	0.27279754685682
C	-3.60683169889008	-1.10281680675798	-1.04572788607723
C	-5.04436250360828	-1.05809253735711	-0.99805387420961
C	-2.93730920400941	-1.11443982007759	-2.30552427612535
C	-3.70204092058068	-1.07978967780749	-3.52398572064675

C	-1.58778943645776	-1.09349926071024	-4.84575947453030
C	-3.02249613458430	-1.07386875979970	-4.78899510528279
C	-0.92526502768447	-1.07171419805386	-6.11365238720920
C	-1.71822235090162	-1.04146497942692	-7.30098604509081
C	-4.76817190694011	-0.95666688361254	6.42749750526420
C	-5.50105907173302	-0.96886504715687	5.25953906402775
C	-3.44026843432926	-1.03527728291692	3.91936741507543
C	-4.86935080604180	-1.00509260925368	3.97932862938517
C	-2.76298901751890	-1.08017160758975	2.65051241152882
C	-3.51925131037165	-1.08524391813297	1.44086899917665
C	-1.41812905296911	-1.16719274091228	0.12248167573024
C	-2.84801775363088	-1.12307321881320	0.17256063551319
C	-0.74454505390751	-1.18290859708209	-1.14513228476725
C	-1.50316683236300	-1.14876211139792	-2.35824529590699
C	0.59825242908493	-1.13274826868931	-3.67452848535437
C	-0.82746470732380	-1.12966453177570	-3.62461691214006
C	1.27104782443200	-1.09864610102103	-4.94604182667322
C	0.52133008336544	-1.07387734361059	-6.16429943440387
C	2.60711536422046	-1.03492991507452	-7.45024643105400
C	1.22908529467153	-1.04480634928797	-7.40430705480705
C	-2.57431391165323	-0.95734299301136	7.59356179938363
C	-3.34547593264981	-0.97764820097230	6.39761028074541
C	-1.23844431976342	-1.03623063755865	5.08959868835640
C	-2.67364085127340	-1.01834342712918	5.13345739976868
C	-0.56584257977438	-1.08018255541807	3.81821912622707
C	-1.32872442833577	-1.11058651289887	2.60267516907954
C	0.77666368457057	-1.16326656613774	1.28911922283653
C	-0.65753858020009	-1.15866392320164	1.33952258502020
C	1.45025673233643	-1.17672888685921	0.02148868987409
C	0.68964563204329	-1.18776937395980	-1.19549708093691
C	2.79550147468613	-1.12775531065175	-2.50665406565457
C	1.36106264457012	-1.15610690376181	-2.45869125500947
C	3.47291238386196	-1.09418137202949	-3.77573889265317
C	2.70628950403168	-1.08414875109354	-4.98991152885487
C	4.80087545461980	-1.03209057344734	-6.28418800493322
C	3.37817945195742	-1.05125846687445	-6.25419291393902
C	-1.19626373565837	-0.97087573783732	7.54759681874882
C	0.95796921272728	-1.01183035156231	6.25702843657788
C	-0.48860981885329	-1.00770586534540	6.30771585113105
C	1.62033020834095	-1.04467983576003	4.98926300157759
C	0.85985745243512	-1.08452393167712	3.76824805760328
C	2.96960538317513	-1.09721291670113	2.44937917568787
C	1.53534708987771	-1.11945340581670	2.50214218823215

C	3.63913956044788	-1.10555948194154	1.18958342000764
C	2.88034903221116	-1.13941494552009	-0.02852140792414
C	4.98931993310803	-1.08158850239008	-1.35009919120598
C	3.55184860848087	-1.12063913463972	-1.29704537256459
C	5.66937945314141	-1.05970447016726	-2.60855256529636
C	4.90203942076080	-1.06585613689520	-3.83586181256875
C	5.53378401819099	-1.03840664136857	-5.11624070254685
C	3.12905838002867	-0.97299059944732	7.39337075824971
C	1.75113510300595	-0.97911466428295	7.44418659726285
C	3.81428117528588	-0.99746733833119	6.14628049236419
C	3.05511597765947	-1.03476726651851	4.93248137764325
C	5.16441783988502	-1.03228747868120	3.62732665310287
C	3.73452472014579	-1.05578463821474	3.66756690408233
C	5.84372430443097	-1.03729879288265	2.34903950424102
C	5.07689542967053	-1.06913227022474	1.14167645954041
C	7.17588240262272	-1.02396175426390	-0.17958831653251
C	5.74509691513952	-1.06065514441819	-0.12930962302206
C	7.82605307618858	-1.00995461375793	-1.44550330898018
C	7.09705804083745	-1.02597249992226	-2.61579943688396
C	5.23564468389897	-0.98335522715878	6.07649823902282
C	5.88470904651291	-0.99903222029777	4.86005080125217
C	7.26832666553521	-1.00504194767788	2.25610123833749
C	7.91335740102395	-0.99927797281851	1.03739457247976
H	-8.89349047062587	-0.93203422443410	1.61934745729870
H	-8.98019282914195	-0.94380504403265	-0.84781502687906
H	-7.84062103480734	-0.97862104139267	-3.02851006716920
H	-6.94909565480052	-0.99531575379260	-4.70685116678715
H	-5.77928640053453	-0.99182872951029	-6.87110851605635
H	-3.68628576954563	-1.00238367285424	-8.17941733971153
H	-7.60411863290346	-0.94522242013019	3.71423719727213
H	-1.22909287382331	-1.02652315261304	-8.28338698377403
H	-5.27817102485442	-0.92863429463785	7.40306307451970
H	-6.59646578866670	-0.94954102598229	5.32683247019375
H	3.13068508533586	-1.01044220281908	-8.41860117028833
H	0.67195127964815	-1.02709361545563	-8.34972047755143
H	-3.09775615597774	-0.92836421225352	8.56191429670789
H	5.31084679643468	-1.00896171923934	-7.25985112554564
H	-0.63908282351742	-0.95164023037256	8.49300541859109
H	6.62916700463358	-1.01958834221445	-5.18363371153494
H	3.71945124800151	-0.94691578719053	8.32250665333108
H	1.26211659010701	-0.95674810307395	8.42655486219092
H	8.92641907278986	-0.98287629141856	-1.47673349707673
H	7.63692367085620	-1.01059731859046	-3.57131832971717

H	5.81260997552457	-0.95754676357947	7.01404423080761
H	6.98212110978170	-0.98440128322130	4.84980117883770
H	7.87356628823358	-0.98188592791631	3.17137608083293
H	9.01315491307036	-0.97252004365244	0.99037568639457
C	-7.72655723642469	3.78086221481178	1.28153376390165
C	-7.07660113250372	3.79508607507362	0.01550569287405
C	-7.81428362636876	3.77053975294176	-1.20134894786041
C	-5.74487298047806	3.80882655862957	-2.51333531359430
C	-7.16945964358576	3.77650864034903	-2.42016248261608
C	-5.06578410399862	3.80397881656597	-3.79173900782283
C	-5.78628994149502	3.77094189660567	-5.02434377658727
C	-3.71608042439946	3.76929132622558	-6.31092736156871
C	-5.13743321181526	3.75533784937294	-6.24090380780671
C	-3.03107484091274	3.74481919883431	-7.55813032654796
C	-5.56968944194655	3.83059890397100	2.44422535172848
C	-6.99736497356956	3.79676684889338	2.45171108899751
C	-4.88984761981951	3.85267847400205	1.18565822223204
C	-5.64582617342754	3.83185784767637	-0.03500433705941
C	-3.54008691371855	3.87695900060927	-1.35424558462523
C	-4.97783628882549	3.84051672220971	-1.30609894556128
C	-2.87076688328830	3.86868469806215	-2.61415505863395
C	-3.63589515335403	3.82739284758215	-3.83222086157040
C	-1.52193402051982	3.81616427452218	-5.15427230342450
C	-2.95670819912744	3.80641525335446	-5.09725458933543
C	-0.85979106015833	3.78326303742540	-6.42214915393479
C	-1.65316122991294	3.75073837516893	-7.60917521779042
C	-4.70057297438241	3.80269267135898	6.11971410223452
C	-5.43367422940940	3.80902453868720	4.95189020204850
C	-3.37303159236249	3.86515324217892	3.61104455445579
C	-4.80214386706254	3.83667815265295	3.67140666662398
C	-2.69584128833700	3.89888155999412	2.34185163251457
C	-3.45238824496543	3.89181606583072	1.13236707800088
C	-1.35100807284705	3.94804201263481	-0.18651066747304
C	-2.78109244672077	3.91072261798795	-0.13626576719743
C	-0.67763002567364	3.93459099055069	-1.45425358604540
C	-1.43651507455642	3.89084904773904	-2.66715817229766
C	0.66443272283922	3.85140544002583	-3.98359162264758
C	-0.76125360195798	3.85589204774713	-3.93338285835392
C	1.33681737889261	3.80733893518786	-5.25508630650268
C	0.58677570112649	3.77889116916424	-6.47307644341098
C	2.67225605674074	3.72810020444536	-7.75926544696070
C	1.29421551360028	3.74187497624762	-7.71307134224804
C	-2.50661966348635	3.80575216448288	7.28540758122102

C	-3.27788654612726	3.82205292523725	6.08947863308321
C	-1.17098218741742	3.86978293002408	4.78098212791086
C	-2.60621172788501	3.85511569851351	4.82508714353267
C	-0.49839859626574	3.90398284782754	3.50935895771273
C	-1.26141357476523	3.92732488663272	2.29364724935427
C	0.84397045577392	3.95418979158163	0.97973911278396
C	-0.59019830366621	3.95904563978204	1.03034502989754
C	1.51733134727936	3.93843399319004	-0.28798358942352
C	0.75654845562282	3.92991033510414	-1.50489730626315
C	2.86177217302706	3.85122198426732	-2.81625715746651
C	1.42751966709421	3.88176205646627	-2.76817235583379
C	3.53883486410604	3.80616956163565	-4.08521979732827
C	2.77200429968382	3.78924625737559	-5.29917973790970
C	4.86630931006555	3.72715953403910	-6.59356596578131
C	3.44362381687677	3.74836428510145	-6.56344274989336
C	-1.12859830213625	3.81583826053864	7.23924251006828
C	1.02553410432751	3.84311715749662	5.94822635707846
C	-0.42105752113493	3.84507343650856	5.99911603211281
C	1.68784243390899	3.86492835626383	4.68021830069846
C	0.92730626397984	3.90099671820250	3.45920947223959
C	3.03692036014517	3.88580881830073	2.13975465553682
C	1.60278634255587	3.92009260450307	2.19272568644932
C	3.70622603519737	3.87410039811399	0.87983811429433
C	2.94719975384154	3.89428168612327	-0.33832550851755
C	5.05562313562967	3.81370842720336	-1.65983100672200
C	3.61823261243802	3.85632254990109	-1.60673967640100
C	5.73537808017389	3.77810710050456	-2.91819512310370
C	4.96790138853156	3.77581544386466	-4.14541823086848
C	5.59938981132594	3.73936618162762	-5.42573167301120
C	3.19651267841144	3.79903445374057	7.08441138269074
C	1.81869780666556	3.81304511282323	7.13543228521098
C	3.88163246432716	3.81155972769436	5.83709562305705
C	3.12253909938296	3.84541924203429	4.62321901829619
C	5.23158066784931	3.81767706198417	3.31779160551522
C	3.80186820550182	3.85127967170329	3.35809053468080
C	5.91066926216862	3.80663200985173	2.03939969966875
C	5.14374845598552	3.82935179995343	0.83192547786301
C	7.24225776032146	3.76152361551278	-0.48917107862146
C	5.81160357988342	3.80373714513412	-0.43903395017189
C	7.89212888592643	3.73316188308777	-1.75501341517771
C	7.16297895777109	3.74063562526489	-2.92533431855567
C	5.30285208672464	3.78742801733303	5.76721512660504
C	5.95185338930025	3.78969136485423	4.55064450290005

C	7.33508673906737	3.76681975885543	1.94654871917019
C	7.97986705609121	3.74629055217498	0.72787947641321
H	-8.82691835682216	3.75371504751693	1.31294907503983
H	-8.91407027734605	3.74371917314105	-1.15414516479754
H	-7.77485152142609	3.75344288382216	-3.33533944740759
H	-6.88370167397503	3.75642763799902	-5.01390456273487
H	-5.71456584594598	3.72969524535139	-7.17835223181490
H	-3.62162510229222	3.71889945745562	-8.48716862274422
H	-7.53707541259826	3.78123689752732	3.40731589189927
H	-1.16430638190105	3.72836882176906	-8.59162592215458
H	-5.21037512798513	3.77941051168756	7.09546013609698
H	-6.52904455041621	3.79006231474189	5.01945821004168
H	3.19553259770783	3.69896229940968	-8.72770376354079
H	0.73687280662378	3.72266913474167	-8.65838520507377
H	-3.03002682021252	3.78112016561750	8.25384802377693
H	5.37613879832288	3.69895377207237	-7.56921396482865
H	-0.57130077623540	3.79814485230635	8.18455980366553
H	6.69478247299168	3.71987480676841	-5.49320270412252
H	3.78691103642253	3.77427244704019	8.01353314688467
H	1.32972966969899	3.79812485534721	8.11791602304852
H	8.99242920724181	3.70276307757607	-1.78611350717965
H	7.70270887222292	3.71576513015451	-3.88077949733977
H	5.87969149300316	3.76379896332175	6.70486446239220
H	7.04912626133964	3.76713349623881	4.54040409191239
H	7.94035346944396	3.75002972439046	2.86191543761945
H	9.07955085616787	3.71487805760122	0.68103215002720
H	-0.31101666467064	1.39270693846077	-0.21047654167554
H	0.40996533090880	1.37863393863430	0.04531796841614

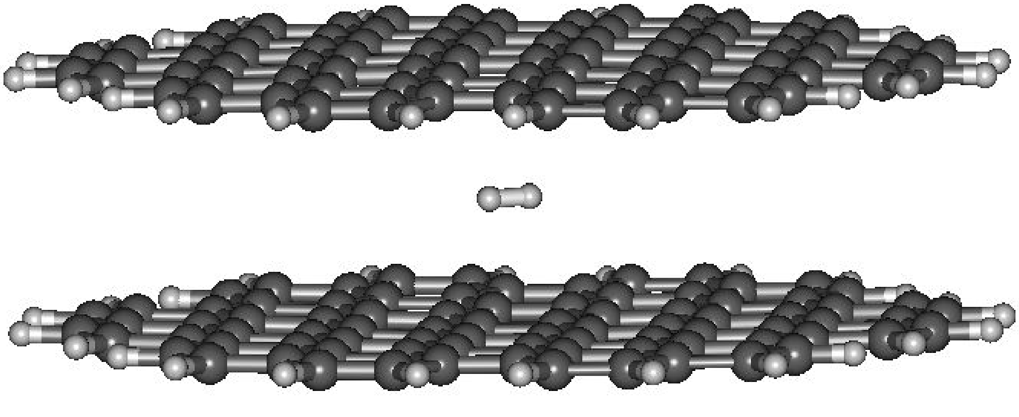

Figure S8. Optimized geometry of D_2 between two graphene sheets: C_{84} D_2 C_{84}, lying horizontally.

9. xyz coordinates of optimized geometry of D_2 inside single-walled carbon nanotubes (2,2), lying horizontally 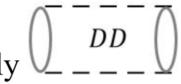

C	-0.38591478042985	-0.52729236232937	-1.42831786245141
C	-0.38606263939189	0.82877029250726	-1.32967775860073
C	-0.21584121199338	0.61815130928879	1.58538206582910
C	-0.21870655567274	-0.73790906594480	1.48530907263365
C	0.82636206754989	1.30860186521785	-0.58995068859088
C	0.90827321737018	1.20881398869569	0.78843395856892
C	0.90580683770801	-1.20667766282497	0.61267089283584
C	0.82758682032965	-1.10725824844381	-0.76600767401359
C	2.26455471131456	0.65942666311008	1.18881178972865
C	2.26145850924914	-0.72298818425312	1.09281252911713
C	2.12480356221897	-0.54513990256647	-1.31910366927780
C	2.12249007887034	0.83892103531278	-1.22137127450967
C	3.53235179108061	-1.21744505016173	0.46338201393260
C	3.45365566375788	-1.11343783338960	-0.91770516794250
C	3.45065853955364	1.33936903070553	-0.73976749143557
C	3.53596949434280	1.23415642639606	0.63595534376540
C	4.71871233369603	-0.53009228995178	-1.47670358204387
C	4.72208122685064	0.85426446601481	-1.39300910372931
C	4.86864549687263	0.65903562049456	1.04629063779571
C	4.85899536752531	-0.73403041060055	0.96917610019187
C	6.04201498745728	1.42972992606214	-0.92029093481928
C	6.12137781384819	1.26603020846599	0.49426261433060
C	6.12093096737489	-1.24709311104137	0.33037328184640
C	6.03859185501470	-1.10810551705760	-1.06139971181629
C	7.41997960103106	0.69354190602272	0.90804428251922
C	7.45621627517761	-0.76276133264113	0.90102755513485
C	7.31879888295257	-0.48087193650629	-1.62704362625332
C	7.28564403309997	1.09870513913881	-1.65499302923014
C	8.73109748294539	-1.28857874003237	0.37260007732309
C	8.66592118510982	-1.03099285802419	-1.18746168486286
C	8.58409326731403	1.51361444678178	-1.08664627095204
C	8.65045768478834	1.30623589044918	0.34927917297966
C	9.94544832350744	-0.40588269621389	-1.75490732529618
C	9.89304672615057	0.99340612185580	-1.68321454094779
C	9.95361796658708	0.78624313711207	0.82233617289853
C	10.00259403683591	-0.63522811532457	0.77682426936163
C	11.18025784715934	1.52759355052751	-1.11715538025594
C	11.20417084701910	1.39747533578301	0.27185934335312
C	11.29947394137593	-1.04673099064286	0.10654330017301

C	11.26910104020354	-0.92448787949860	-1.27366630093047
C	12.53894247101815	0.88416484335016	0.74955189409276
C	12.59559861535041	-0.49375665408613	0.64747269412853
C	12.53387497374528	-0.31109910895980	-1.80331425965617
C	12.48434041757223	1.07097547280397	-1.70154927761621
C	13.90139059576232	-0.92338688948422	0.05025581753358
C	13.86555544250560	-0.81471921165566	-1.33260629569840
C	13.77938228804668	1.59910616999906	-1.15387733572552
C	13.80948702802690	1.48957200112472	0.22719528090848
C	15.16192897300997	-0.21014165500191	-1.84119122400096
C	15.11300425625951	1.16940330649910	-1.73616294746266
C	15.16809952699609	0.98766463788412	0.67816398920481
C	15.22170601170541	-0.39186831364724	0.57375572694068
C	16.26685033533848	1.94238880276675	-1.17301278708166
C	16.29657119431593	1.84109071924502	0.18261091365727
C	16.40616221023499	-1.07561749531262	-0.04063063868161
C	16.37272590051103	-0.97377371620353	-1.39607497068772
H	-1.14641879754751	-1.10826556098913	-1.97435508036853
H	-1.14533306812368	1.48316572706269	-1.78722316498289
H	-0.91286777923091	1.19395519130256	2.21513464517259
H	-0.91901731498673	-1.39708466629931	2.02291844454121
H	16.96370293000212	2.55865073170149	-1.76336143956010
H	17.02021810086035	2.36555488111393	0.82705617792222
H	17.16990453443055	-1.63876863911031	0.51940767512377
H	17.10753671260384	-1.44202268365984	-2.07050772265433
H	7.28727656138256	-0.02408766001248	-0.57216831063606
H	8.73828058445372	-0.06230040292479	-0.57092320077235

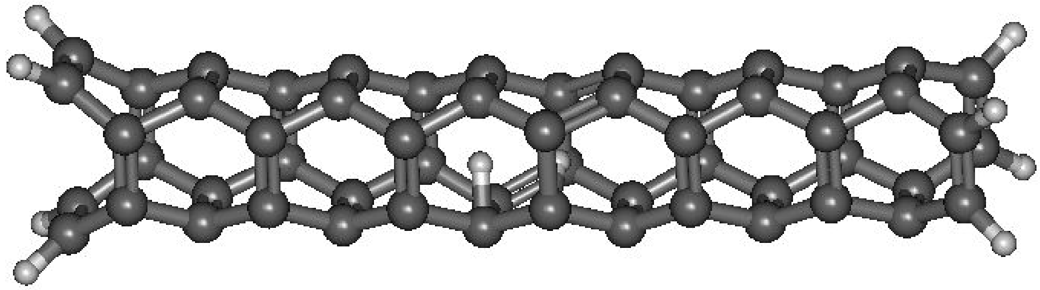

Figure S9. Optimized geometry of D_2 inside single-walled carbon nanotubes (2,2), lying horizontally.

10. xyz coordinates of optimized geometry of D_2 inside single-walled carbon nanotubes (3,3), lying horizontally 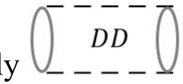

C	1.56072940443449	1.55697523148879	-0.07587764020966
C	0.34864466815976	2.17623404334699	-0.06366190344136
C	-2.12141946892700	0.57330382700686	-0.03028918097216
C	-2.05260742849288	-0.78616811367014	-0.02791283687590
C	0.57011954665608	-2.12321099618043	-0.05529289721945
C	1.71255310112141	-1.38327058499333	-0.06974964999659
C	-0.45630389000566	2.02420635605862	1.16553718235437
C	-1.62963092850554	1.25967416885413	1.18150737363178
C	-1.49399968046408	-1.41508693963599	1.18624627334554
C	-0.24625487369307	-2.04951839576835	1.17333087176086
C	2.00102878253501	-0.59286009197259	1.14428052141920
C	1.92790094117203	0.80531682521459	1.14118788036324
C	-1.98005959327879	0.62615318160170	2.45037257583194
C	-1.90514558214068	-0.81512358369969	2.45315454881361
C	0.50200426114948	-2.04473967271776	2.42834495873588
C	1.71059678861177	-1.25621063847271	2.41282396729568
C	1.57431949559356	1.44334525070484	2.40696035351705
C	0.28844085137405	2.09713136772518	2.42022235337810
C	-1.51774237062673	-1.44317292904390	3.68052646103589
C	-0.21394345987967	-2.10564159671463	3.66711751438906
C	2.08431032769935	-0.61444698395165	3.63718338042420
C	2.00880216826722	0.84651656320135	3.63399314696876
C	-0.43054399097510	2.09183966773772	3.65906074694508
C	-1.65551340845254	1.29255645885817	3.67541678399017
C	0.53366486664452	-2.03569120016058	4.89235566229576
C	1.73454181544085	-1.25163547028373	4.87660717970571
C	1.59910571088294	1.45184719606873	4.87095878568928
C	0.32108169974560	2.10118668534981	4.88393990519866
C	-1.95026287847657	0.62743087742540	4.91429634747676
C	-1.87667207388966	-0.80482126717215	4.91690954846021
C	2.06733995016981	-0.58931640045280	6.12941825950027
C	1.99447139799092	0.82981241996401	6.12664774154425
C	-0.39415428816221	2.04971468413983	6.15097737013961
C	-1.58344222668191	1.27262352067120	6.16664841972838
C	-1.45106113381460	-1.40682766262922	6.17195328382634
C	-0.18410870814211	-2.04991815045862	6.15903127802669
C	1.65377432849880	1.47909628428227	7.38046335136062
C	0.35218704508644	2.14484870643704	7.39365512751122
C	-1.95329624127985	0.64565072611144	7.42389586836042

C	-1.87830773402066	-0.81329033065472	7.42679603268440
C	0.56852549352711	-2.06252177558164	7.40130157803088
C	1.79327006736106	-1.26568354216045	7.38539447284497
C	-0.39028861925094	2.15296282445844	8.62049944504725
C	-1.63725178439561	1.34243379426061	8.63707889791391
C	-1.49118733752291	-1.46557896712493	8.64306681490629
C	-0.16729332182129	-2.14071532475245	8.62911683500980
C	2.18958616689986	-0.61115971876047	8.59823172045007
C	2.11377819531020	0.87345362294361	8.59523067563533
C	-1.90159156478174	0.64588731216276	9.87264592823064
C	-1.82607019574915	-0.79261229715436	9.87528539105510
C	0.59379707466124	-2.03041435195967	9.85088620466424
C	1.80088939507533	-1.24483046081035	9.83512027025903
C	1.66027628497170	1.46889329667772	9.82878880125397
C	0.37850711799368	2.12354899713923	9.84201690867040
C	-1.39966582852121	-1.39739034996876	11.13197837678130
C	-0.12626460699809	-2.04537194756174	11.11909303324003
C	2.13454664036694	-0.57993460673524	11.08932567721022
C	2.05929918651352	0.84728364211206	11.08617530162425
C	-0.33838818229648	2.07285221080714	11.11117273056872
C	-1.53541936665942	1.29264083241806	11.12723966456308
C	0.62256962616452	-2.04178325091398	12.35056356483492
C	1.84566729738846	-1.24458253568506	12.33449159011778
C	1.70696370530042	1.48591543780111	12.32888576196158
C	0.40613011339083	2.14779823140659	12.34258100897551
C	-1.88817860903590	0.66002804438564	12.37281612539106
C	-1.81220063480025	-0.79810801391755	12.37562965239780
C	2.18110799583794	-0.58471178749712	13.55663926045260
C	2.10561804127181	0.86642184530988	13.55352692071650
C	-0.30964444011680	2.10057836529579	13.57863143728612
C	-1.52648856579552	1.30672209155288	13.59457520822655
C	-1.38960050712941	-1.40201936658696	13.59983035731365
C	-0.09494783957197	-2.06056841456295	13.58652697163734
C	1.69282686608236	1.46592828824265	14.81309463395944
C	0.43922490186891	2.10377361422319	14.82614794253487
C	-1.81484044093842	0.64186697205187	14.85584887241622
C	-1.74177702538484	-0.76303437557116	14.85851963213893
C	0.64976030836904	-1.98595214939587	14.83410769888988
C	1.82859973333447	-1.21751662747681	14.81827105360936
C	-0.37186335397780	2.16273750583024	16.05696170941138
C	-1.51455836175822	1.42138478962595	16.07193520543041
C	-1.36455516508565	-1.50306834928093	16.07751061964470
C	-0.15159180846726	-2.12266873438150	16.06539480954431

C	2.30466032432333	-0.52797893784363	16.03243064627759
C	2.23589745914240	0.83247889310304	16.02977606537635
H	2.19025007364362	1.52795451242794	-0.97907916675123
H	-0.05680728416082	2.67799910851846	-0.95633363174264
H	-2.42931632879442	1.13841394710961	-0.92409577852277
H	-2.30208550783770	-1.38245485305471	-0.91960157456966
H	0.21713311627080	-2.66636532869577	-0.94599333054868
H	2.33558704073378	-1.29291494999179	-0.97340372799250
H	-0.01058043918181	2.68818962691270	16.95486864268958
H	-2.12598706483276	1.31857527922919	16.98208123645282
H	-1.98302949427793	-1.45845672209575	16.98761044247774
H	0.25936061764039	-2.60773654904784	16.96466330580894
H	2.59405045227136	-1.09105898662111	16.93358343144697
H	2.46647546705228	1.42521724298504	16.92879799678981
H	0.11289272101800	0.06579941121894	8.19525926251832
H	0.08504200403416	-0.01036249863544	8.93503246844462

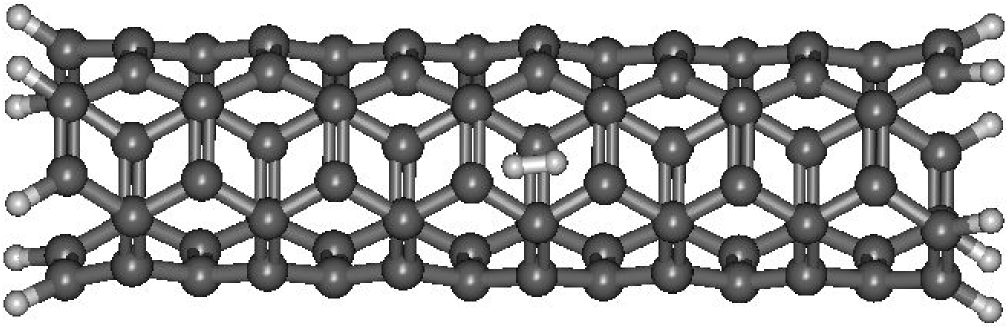

Figure S10. optimized geometry of D_2 inside single-walled carbon nanotubes (3,3), lying horizontally.

11. xyz coordinates of optimized geometry of D_2 inside single-walled carbon nanotubes (4,4), lying horizontally 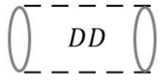

C	2.86405373244189	0.05366226502825	-0.01511538687405
C	2.50739497120436	1.38548553054847	-0.01504628406737
C	-0.05290713189099	2.86317515751709	-0.01511648529380
C	-1.38472875628745	2.50651242201696	-0.01504319238139
C	-2.86234522945780	-0.05383157237112	-0.01512253530475
C	-2.50568530368691	-1.38565444956785	-0.01505215763585
C	0.05461538960475	-2.86334539441667	-0.01511509230432
C	1.38643697514563	-2.50668239657785	-0.01503891047284
C	1.98370402571375	1.98516533672465	1.19189125005549
C	0.72833956216905	2.70981143761268	1.19175062608040
C	-1.98439291321829	1.98279529174048	1.19189368282405

C	-2.70900285997820	0.72740872916680	1.19174984655037
C	-1.98199841949736	-1.98533348747002	1.19188698922202
C	-0.72663448394816	-2.70998033706163	1.19175028687610
C	1.98609720878143	-1.98296373312379	1.19189880207893
C	2.71070647772682	-0.72757752220793	1.19175625715649
C	0.00681442923415	2.77929064466744	2.42386356728256
C	-1.39393584196056	2.40337631790939	2.42395102342347
C	-2.77847327624045	0.00587856980265	2.42386033770682
C	-2.40257597581718	-1.39487355935643	2.42394422907326
C	-0.00511199909029	-2.77945923236540	2.42386474394080
C	1.39563813928809	-2.40354470192730	2.42395447869388
C	2.78017347940519	-0.00604725010126	2.42386671147415
C	2.40427801240489	1.39470511468746	2.42394963867392
C	-1.95731702730142	1.95494773318243	3.66832828614519
C	-2.67203590899378	0.71793134115087	3.66826055008450
C	-1.95417385366095	-1.95827352637911	3.66832201068670
C	-0.71717200383136	-2.67301922095415	3.66826193375149
C	1.95901536966357	-1.95511544330379	3.66833252908274
C	2.67373238557581	-0.71809886371225	3.66826683759612
C	1.95587284579057	1.95810513934236	3.66832614193414
C	0.71887161864631	2.67285128723778	3.66826229842553
C	-2.77928184302462	-0.00059917617704	4.91347167716030
C	-2.40659540283931	-1.38938784932546	4.91347008678152
C	0.00135364281446	-2.78026567715641	4.91347868077891
C	1.39013218687682	-2.40754342931441	4.91347826207811
C	2.78097397408182	0.00043151716497	4.91347840295816
C	2.40828903234378	1.38921843577403	4.91347539729820
C	0.00034328188620	2.78009832756974	4.91347732634300
C	-1.38843551317418	2.40737572352353	4.91347562195440
C	-1.97421970257311	-1.97205583096720	6.14772076811487
C	-0.71965039001263	-2.69650061853108	6.14760051638612
C	1.97278112166432	-1.97514530149365	6.14772624716432
C	2.69720405493166	-0.72056588660780	6.14760217956078
C	1.97591195186860	1.97188676536357	6.14772450422809
C	0.72134354627970	2.69633371231786	6.14760148857511
C	-1.97108865462665	1.97497681720156	6.14772230088362
C	-2.69551467865688	0.72039883038911	6.14759601404171
C	0.00265467616460	-2.78708557730095	7.38384032718248
C	1.39208680611860	-2.41443342343797	7.38394568690978
C	2.78779986980962	0.00173965056990	7.38383981619648
C	2.41518846344396	1.39118136098428	7.38394452561619
C	-0.00096538307501	2.78691768653674	7.38383925302169
C	-1.39039723371940	2.41426598458444	7.38394216426747

C	-2.78611206800761	-0.00190663852172	7.38383515417192
C	-2.41349802765499	-1.39134949137361	7.38394085501100
C	1.97227541467131	-1.96919124132547	8.62674743412633
C	2.69037964706992	-0.72292732651197	8.62662022988068
C	1.96995935923058	1.97138106750900	8.62674843618193
C	0.72369920385220	2.68949855647597	8.62662329957707
C	-1.97058923477569	1.96902721469753	8.62674432006609
C	-2.68869429223027	0.72276221824397	8.62661623803712
C	-1.96826943358128	-1.97154345945296	8.62674778880013
C	-0.72201221658376	-2.68966258414215	8.62662290393256
C	2.79214995860936	-0.00244270962608	9.86326505781959
C	2.41603901957018	1.39656933731530	9.86334828778494
C	0.00321185606445	2.79125479276456	9.86326716179525
C	-1.39579692512854	2.41511727416265	9.86334540313797
C	-2.79046540898900	0.00227866109425	9.86326181288134
C	-2.41434989430352	-1.39673722713520	9.86334624657085
C	-0.00152488787086	-2.79141835150736	9.86326853915681
C	1.39748414255090	-2.41527980735924	9.86334746963457
C	1.96553921242739	1.96540899126256	11.09742417576157
C	0.72071900446144	2.68494028838482	11.09743923103116
C	-1.96463610615811	1.96461239164231	11.09741811872222
C	-2.68415868841758	0.71978833855177	11.09743247948352
C	-1.96385216963606	-1.96557480578876	11.09742417481542
C	-0.71903152949040	-2.68510396478316	11.09744059217875
C	1.96632152634621	-1.96477498867819	11.09742064696186
C	2.68584283608075	-0.71995171930709	11.09743490436465
C	-0.00120191394565	2.76820064895692	12.34253469936019
C	-1.38099210829117	2.39755237484234	12.34249894486269
C	-2.76742923240261	-0.00212820560326	12.34253002232931
C	-2.39678632784410	-1.38191965021867	12.34250198737543
C	0.00288853354961	-2.76836289324272	12.34253611603088
C	1.38267770142148	-2.39771462487682	12.34250078447132
C	2.76911378244969	0.00196406309116	12.34253217072433
C	2.39847120585114	1.38175508281871	12.34250306213095
C	-1.95946169626824	1.96872386387852	13.58688834031580
C	-2.68479132446731	0.71311846561719	13.58696184572012
C	-1.96795572257191	-1.96037944374674	13.58689604678435
C	-0.71235244699136	-2.68571227064020	13.58697067468466
C	1.96114672885907	-1.96888678259081	13.58689002428070
C	2.68647585381469	-0.71328158304576	13.58696393461925
C	1.96964029007955	1.96021603019620	13.58689684311071
C	0.71403778370941	2.68555020161998	13.58697048278688
C	-2.80522685952451	-0.00151919121213	14.81912947151404

C	-2.43016259419603	-1.40195921734339	14.81902909341774
C	0.00229104214824	-2.80614094743484	14.81913644037465
C	1.40273671093035	-2.43109592321119	14.81902604902885
C	2.80691058425852	0.00135665133882	14.81913128711583
C	2.43184643217031	1.40179652749547	14.81903047197345
C	-0.00060650174513	2.80597911895361	14.81913566004172
C	-1.40105140506663	2.43093318347064	14.81902463681959
C	-1.98643574218066	-2.06303198146458	16.02573084345884
C	-0.79207606071132	-2.75209098482409	16.02581992191232
C	2.06383147760359	-1.98738272480793	16.02572216068979
C	2.75288734133804	-0.79302105863606	16.02581000970206
C	1.98811949676880	2.06287025895932	16.02573147563429
C	0.79375990974778	2.75192917998708	16.02581953678324
C	-2.06214848177870	1.98722107193016	16.02571970879179
C	-2.75120478967073	0.79285979408163	16.02580740559528
H	3.09958840102861	-0.44134311651798	-0.96945556536546
H	2.46415176212734	1.93189674266253	-0.96940222201008
H	0.44208994038365	3.09873102282718	-0.96945568083904
H	-1.93115152713973	2.46328933948279	-0.96939366520712
H	-3.09787693185050	0.44117437410248	-0.96946336820623
H	-2.46243756202360	-1.93206468618737	-0.96940815813317
H	-0.44037990326082	-3.09890393511344	-0.96945487977045
H	1.93286200885449	-2.46346220715645	-0.96938810563393
H	-2.50292373673081	-1.87905591588090	16.98000512513628
H	-0.37450611219024	-3.10703606937801	16.98020660461066
H	1.87987961170721	-2.50388493607000	16.97999360648454
H	3.10785651988137	-0.37546216450570	16.98019282623037
H	2.50460691967197	1.87889496390791	16.98000604758096
H	0.37618924245932	3.10687488322526	16.98020576315629
H	-1.87819829647515	2.50372427084929	16.97999081285482
H	-3.10617577130475	0.37530173096733	16.98018982848341
H	0.00085926489199	-0.00017181240522	7.56035279218822
H	0.00086576030098	0.00001099515294	8.32457086212629

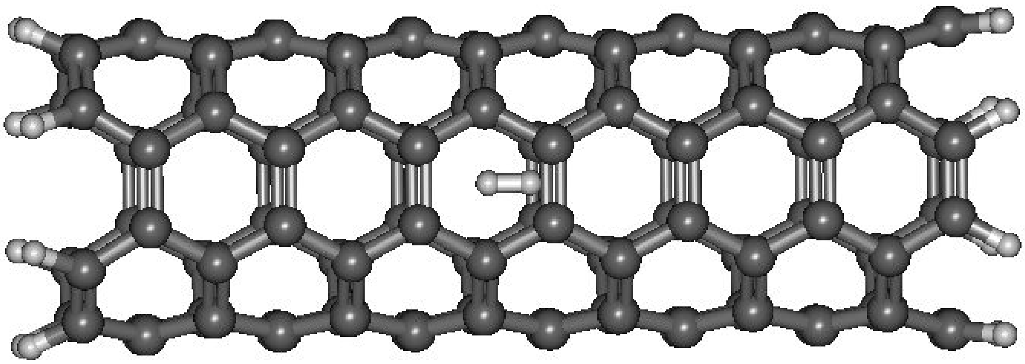

Figure S11. Optimized geometry of D_2 inside single-walled carbon nanotubes (4,4), lying horizontally.

12. xyz coordinates of optimized geometry of D_2 inside single-walled carbon nanotubes: (4,4), positioning vertically 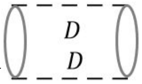

C	-0.07264048172031	2.77967688210605	-0.68939964640325
C	-0.09749049426734	2.77887758920337	0.68904351711052
C	-0.08293134763895	0.68799123330570	2.77863061848364
C	-0.04881568713133	-0.69022585976127	2.77855461030232
C	0.04001061200498	-2.77778609549910	0.68818036245503
C	0.06491672490958	-2.77730213057470	-0.69025544519007
C	0.05145056622848	-0.68722163954294	-2.77941942170302
C	0.01729351685924	0.69099313066224	-2.77891145937656
C	1.10471815735726	2.45807939063254	1.42550533431476
C	1.11197281838048	1.43272820275699	2.44981766424858
C	1.18149794499878	-1.37480549275334	2.44948881711729
C	1.22502928689776	-2.39877051533643	1.42469923065832
C	1.27576654653377	-2.39750419217033	-1.38310078759134
C	1.26921666865600	-1.37220836286281	-2.40745813661794
C	1.19962235603822	1.43530313062495	-2.40673422385277
C	1.15536846976638	2.45962631248433	-1.38233096599721
C	2.35588954414270	0.78429804855854	2.72547057217344
C	2.39176822758018	-0.66567773735390	2.72527462777007
C	2.47511533358159	-2.62204589484669	0.76812566150723
C	2.50129715455442	-2.62136733126401	-0.68192542892491
C	2.48863135374270	-0.66267131146951	-2.63833302018355
C	2.45271761360414	0.78722184854488	-2.63798586276874
C	2.36829741144589	2.74397625331520	-0.68122312974971
C	2.34217473799359	2.74317675098018	0.76893990346182
C	3.65695398174646	-1.29476261216850	2.46017316937772

C	3.69989580315354	-2.30473564780901	1.45068230081844
C	3.74993127441706	-2.30330047929521	-1.31948520308358
C	3.74347282171668	-1.29216290286434	-2.32867448554499
C	3.67482615236765	1.47792700828639	-2.32816323023220
C	3.63106660355565	2.48795090854345	-1.31877488128539
C	3.58109641438434	2.48642809649742	1.45139635419204
C	3.58833949645259	1.47531345331746	2.46059754697168
C	4.96362582456662	-2.56375464619491	0.80693412464479
C	4.98959360491199	-2.56298719791353	-0.63071781502588
C	4.97700728559054	-0.59476970037866	-2.59443438467094
C	4.94139851950358	0.84261916017424	-2.59413128840818
C	4.85629318826749	2.80844973450801	-0.62995006989051
C	4.83039591022755	2.80761395972126	0.80759785232299
C	4.84447873521117	0.83958352426169	2.77127366760567
C	4.88008028080013	-0.59774693303660	2.77105362538420
C	6.22881920440419	-2.26582560248983	-1.28317972129170
C	6.22235320737224	-1.23924374743043	-2.30554741196450
C	6.15324706724619	1.54793606704297	-2.30500508222512
C	6.10872259535903	2.57308326524622	-1.28220203141340
C	6.05846482405675	2.57130522196562	1.50440770306945
C	6.06601155844343	1.54512865891301	2.52693142908052
C	6.13511141036198	-1.24172528238798	2.52645127049195
C	6.17844064035395	-2.26726836457333	1.50406218634750
C	7.44616164987403	-0.53400066638275	-2.55649827824968
C	7.41052769022256	0.90465227523681	-2.55644018718210
C	7.32498018844931	2.88119454902329	-0.58643623020660
C	7.29904389455005	2.88038553984571	0.85353013965342
C	7.31337443933234	0.90157533859723	2.82308007857841
C	7.34899191786358	-0.53704185651060	2.82292533598439
C	7.43304557897956	-2.51318376154566	0.85286683979052
C	7.45902858597648	-2.51240123049759	-0.58706424221542
C	8.63102094844245	1.61171453596806	-2.25439222140381
C	8.58748287923501	2.63179676538102	-1.23967060480255
C	8.53701899067193	2.62985395473488	1.55173344989815
C	8.54381244480381	1.60899292681626	2.56642814954117
C	8.61318529067932	-1.18199589536202	2.56623085809094
C	8.65711078212265	-2.20213673895811	1.55156251020776
C	8.70753270954290	-2.20061069135898	-1.24037850369446
C	8.70022716607282	-1.17912281580217	-2.25435755736826
C	9.80381787875847	2.94557618747831	-0.54600471731156
C	9.77761535026473	2.94486132391018	0.90319692057382
C	9.79063428758409	0.96837461687557	2.87041246891548
C	9.82656999206463	-0.47992387976992	2.87021023012776

C	9.91182622647806	-2.45300417167045	0.90234306128023
C	9.93795857248699	-2.45212339115326	-0.54634825607130
C	9.92399859587772	-0.47690096695141	-2.51355482951938
C	9.88812698131000	0.97128482201348	-2.51324540235400
C	11.00616584150274	2.68362282359701	1.59034628971728
C	11.01282604968835	1.66483385880204	2.60471172817536
C	11.08197784884662	-1.11448545497260	2.60445800321315
C	11.12602620853210	-2.13187197407208	1.58968747649963
C	11.17628209303862	-2.13043791071326	-1.18896376624490
C	11.16903199299657	-1.11190516693132	-2.20349727292618
C	11.09996054511030	1.66740576719049	-2.20293563026722
C	11.05649845234073	2.68512599561517	-1.18840129615760
C	12.26929200696197	1.02016887826653	2.89470706163284
C	12.30481944135253	-0.40816745859152	2.89456887220376
C	12.38974544283574	-2.36784843850597	0.93722056255635
C	12.41557387852815	-2.36707584257031	-0.49131291332388
C	12.40159872097009	-0.40528124555412	-2.44834666375730
C	12.36610830431227	1.02307721657638	-2.44805024098601
C	12.28255585065008	2.98278897570995	-0.49066010642103
C	12.25668397917750	2.98206099246764	0.93784497146710
C	13.57011071846672	-1.04658223528172	2.65367724282236
C	13.61459126595971	-2.07199627828033	1.62926859390965
C	13.66470882290084	-2.07047980127179	-1.13813732109173
C	13.65740205117293	-1.04404769749834	-2.16247001915504
C	13.58860448401862	1.72347354433419	-2.16214005566813
C	13.54482384853388	2.74864686497912	-1.13746483011763
C	13.49466153022002	2.74736953290481	1.62989764248529
C	13.50126852029423	1.72094146692641	2.65421253409238
C	14.86572365668933	-2.34224219560421	0.99257870419198
C	14.89193148151149	-2.34146553292083	-0.45672936043312
C	14.87788900513040	-0.35402523212658	-2.44173192636756
C	14.84189783313246	1.09510195294051	-2.44155242565476
C	14.75712755986200	3.07979191814046	-0.45595731074683
C	14.73087057058160	3.07924234593320	0.99317671816334
C	14.74364435253855	1.09214783481163	2.97794564823559
C	14.77968134034132	-0.35685202638408	2.97752738776312
C	16.10531049097713	-2.05234364633257	-1.18781372374458
C	16.09838705035497	-1.07697092040598	-2.16220511876042
C	16.02494200539468	1.87792607289045	-2.16214495761281
C	15.98330161674539	2.85211594740894	-1.18745440599470
C	15.92966127528604	2.85102963451661	1.76869169609842
C	15.93590789395122	1.87570841746369	2.74307340021213
C	16.00947117777573	-1.07978711647162	2.74212834175775

C	16.05178964197446	-2.05414107890883	1.76761679451621
H	-1.01896075773826	2.85572945479829	-1.24616860796330
H	-1.06326143914499	2.85407670534900	1.21147134971070
H	-1.05163073038444	1.20389827399698	2.86078429584896
H	-0.99082799124661	-1.25335508742372	2.86091380743045
H	-0.92105568123343	-2.89947420755430	1.21044015452228
H	-0.87670036513678	-2.89828665856536	-1.24695926718323
H	-0.88709398297190	-1.25017640999243	-2.89529502891219
H	-0.94794302219595	1.20690548349978	-2.89457985561885
H	17.06500441520032	-2.47910942151887	-0.85896488804974
H	17.05264604136379	-0.74087462340343	-2.59543011407174
H	16.99462211339420	1.59081017547563	-2.59641470989033
H	16.92034130848581	3.32673444334737	-0.85884067898440
H	16.87791245612641	3.32683648704458	1.47583749166304
H	16.88903232748044	1.58859031597443	3.21258551202733
H	16.94718712983131	-0.74445489108020	3.21064404480160
H	17.02270715696044	-2.48132690192116	1.47410760023079
H	7.98720487828922	0.58371535873751	0.13919820963982
H	7.99988481162908	-0.17623720643451	0.15382333173911

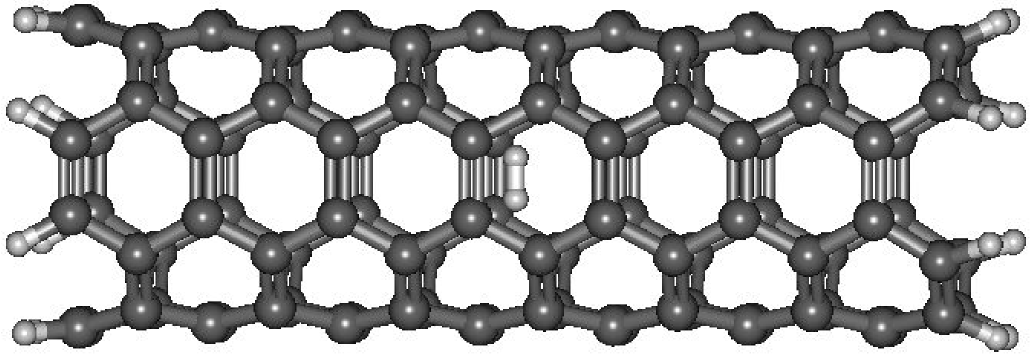

Figure S12. Optimized geometry of D_2 inside single-walled carbon nanotubes (4,4), positioning vertically.

13. xyz coordinates of optimized geometry of D_2 inside fullerene C_{60}

C	2.24012681571489	0.60857756821855	2.69876681118312
C	3.14997790643912	0.17373172821585	1.64606562540188
C	1.34389961005653	-0.30994586936248	3.27581642135444
C	3.13389309152585	-1.16391895299711	1.20953322709249
C	3.23450806395198	-1.46821789675261	-0.21261273440185
C	3.26743489441634	1.25552341810164	0.67639909475379
C	3.36425474272479	0.96204781284419	-0.69664299369209
C	3.34722265768649	-0.42364859198334	-1.14913867485215
C	-0.45942388547246	1.39270976109809	3.24756370735346
C	0.46826957988528	2.34427507347978	2.64853170884778

C	-0.02949076570960	0.08905409307714	3.55631158976350
C	1.79516711752459	1.95977296239163	2.37966241698254
C	2.43011872931003	2.35963129621955	1.12935157048819
C	-0.27038209080148	3.14303046282276	1.67870567959242
C	0.34240439501854	3.52871966370613	0.47242764086373
C	1.71623817640564	3.13019453431837	0.19285378946620
C	-2.16204990994011	-0.84566090792317	2.69835850329632
C	-2.60734424880742	0.50489741925212	2.37834227300868
C	-0.89549193397512	-1.04941455829414	3.27623040164854
C	-1.77109075678199	1.60401269463018	2.64806936403634
C	-1.65410016756882	2.68521345304877	1.67802610501680
C	-3.35656934832845	0.44802559813602	1.12945604917683
C	-3.24374328552039	1.49246538233832	0.19296883485119
C	-2.37808565734185	2.63136842017461	0.47270039449557
C	-0.51432383259338	-3.01366939198825	1.80964270610983
C	-1.82666638660218	-2.80264320981154	1.21058914239080
C	-0.05694633840718	-2.15262751714388	2.82418815425174
C	-2.63624008992017	-1.73771165456299	1.64742067096184
C	-3.37465586717753	-0.93776281248607	0.67757385661833
C	-1.72646698247129	-3.10589121818315	-0.21188170760602
C	-2.43967400512695	-2.33435516851219	-1.14796044059393
C	-3.27765912125589	-1.23072616671682	-0.69545395635676
C	2.20565103797684	-2.11464291602096	1.80798241827591
C	1.73272808704191	-3.00677534879370	0.75723271933460
C	1.32700592921296	-1.69558708052640	2.82391373492174
C	0.39639743808879	-3.44695831739314	0.75725862955916
C	-0.35262783291502	-3.50453565141203	-0.49165657474899
C	2.36784900730225	-2.60646921836484	-0.49225984811944
C	1.64441098779061	-2.66133292851820	-1.69788817081758
C	0.26032168159158	-3.11809417465872	-1.69758667286554
C	-2.25034556853947	-0.58372530019693	-2.71772639196604
C	-1.80501990000541	-1.93465006785761	-2.39823551574188
C	-0.47831938204195	-2.31930719804018	-2.66724014278393
C	-1.35435681448134	0.33470687561657	-3.29523151573530
C	-2.21661857961883	2.14006180653770	-1.82830933405962
C	-3.14451029452605	1.18892488706226	-1.22935427418652
C	-3.16061139227137	-0.14892058148789	-1.66537010641000
C	-1.33762407981193	1.72069769898179	-2.84402569429479
C	0.50403073146078	3.03763570690364	-1.82883513870150
C	-0.40663432409833	3.47182318556707	-0.77676945253783
C	-1.74288255064431	3.03144189494114	-0.77691224058557
C	0.04661006434281	2.17731576429936	-2.84397210372756
C	2.15144974194408	0.86971736212201	-2.71734857018195

C	2.62518073400272	1.76133091943243	-1.66610476004724
C	1.81605447168034	2.82635701638623	-1.22944739664578
C	0.88520574577160	1.07394255466447	-3.29575502732500
C	0.44920013385927	-1.36779050921730	-3.26606870874942
C	1.76113529836659	-1.57960973732628	-2.66751116172770
C	2.59812684835317	-0.48068536500629	-2.39854725622516
C	0.01910658612510	-0.06441542454290	-3.57569705342106
H	-0.28805843165760	-0.25904531919592	0.20788300656074
H	0.36547651884234	-0.19626295931138	-0.19169562855023

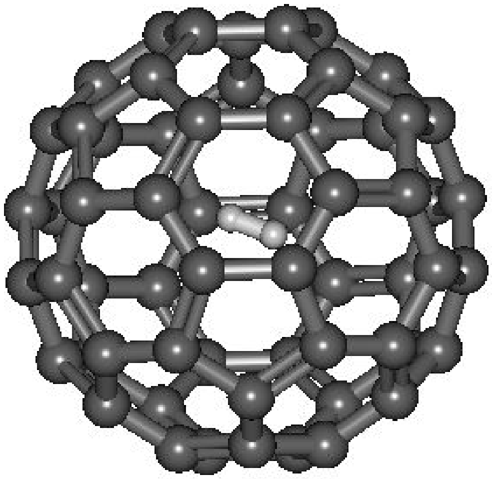

Figure S13. Optimized geometry of D_2 inside fullerene C_{60}.

14. xyz coordinates of optimized geometry of D_2 inside fullerene C_{40}

C	-1.74194869228574	2.45493589601656	-0.70224158054069
C	-0.45771843639034	2.97509589995444	-0.70169372593865
C	-2.10159280936938	1.30995426751252	-1.53301348134523
C	0.59842967506631	2.40694510768351	-1.53304550514219
C	0.30554873196900	1.22412719468577	-2.26798034110461
C	-1.06626569326223	0.66663364434397	-2.26754361350688
C	-1.89226804783407	-1.59703357514400	-1.53281956022709
C	-0.95902017520098	-0.81066511197638	-2.26648266697120
C	0.03428407893134	3.01164493885129	0.69927374314863
C	1.38009673672574	2.68097213502502	0.69893489208159
C	1.79970344074308	2.42103279021739	-0.70320778346295
C	2.69180391795506	1.35989975681975	-0.70332579073573
C	2.47565250134235	0.17934860930079	-1.53362728589429
C	1.25943591323659	0.09180931455169	-2.26795699722392
C	-0.47095907138606	1.16771684869311	2.26458004494979
C	0.96612004253858	0.81238217993761	2.26311060858380
C	-0.92954224715878	2.29637709036735	1.52978231555537

C	1.89951754095942	1.59848303275607	1.52925332255959
C	2.87768768648980	0.90239589810419	0.69839893956749
C	1.07328290169809	-0.66492390537612	2.26389047898519
C	2.10869182651381	-1.30833518801472	1.52953685670515
C	2.97751019306798	-0.47991815630108	0.69896988675314
C	-2.85175046240158	0.96056986727634	0.69966940754543
C	-2.46844172526429	-0.17771383309595	1.53012233110133
C	-2.11900936199276	2.13667548277606	0.69933279263281
C	-1.25241399157353	-0.09006369366958	2.26481095970634
C	-0.29847040268093	-1.22239883287409	2.26447342727493
C	-2.68453688074592	-1.35840364169442	0.69975575701282
C	-1.79245145782116	-2.41944502802120	0.69972915066701
C	-0.59128098094501	-2.40530865903179	1.52958178767091
C	0.46487002490553	-2.97354243529201	0.69837434889246
C	1.74906814710013	-2.45327658472721	0.69888747799608
C	-2.87034724826561	-0.90110158351512	-0.70199880901696
C	-2.97025661071245	0.48136150179934	-0.70241590094890
C	2.85900131221753	-0.95896914690168	-0.70317556822852
C	2.12609143034902	-2.13496047524927	-0.70267552878014
C	0.47797141972798	-1.16584930952040	-2.26755723130311
C	0.93658172812384	-2.29455525520164	-1.53298368387504
C	-1.37289052318826	-2.67946742518893	-0.70239698212368
C	-0.02712149162024	-3.00996374822626	-0.70258525451837
H	-0.35525213712280	-0.11593505438130	-0.04826184747805
H	0.35578919756090	0.12552318673037	0.04691460897636

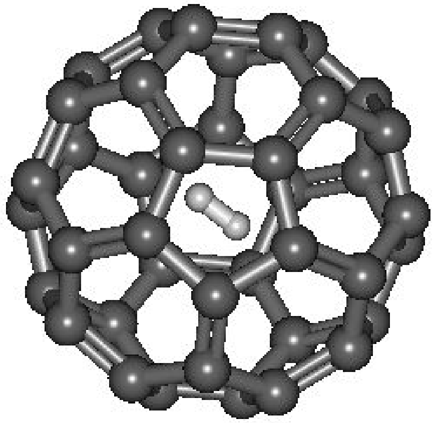

Figure S14. Optimized geometry of D_2 inside fullerene C_{40}.

15. xyz coordinates of optimized geometry of D_2 inside fullerene C_{20}

C	-1.89989505568325	-0.40624239820374	0.60055181991706
C	-1.96785323815311	0.18207362024452	-0.71895346143827
C	-1.20789779679963	0.53217059146972	1.49336758006780

C	-0.81447894068018	1.71695254881588	0.71720809043290
C	-1.27341550933391	1.55096943105966	-0.64630310553675
C	0.00750222682102	-0.11769150049601	1.99371857816703
C	1.17031814353361	0.65768470629495	1.54384820036688
C	0.67978927656876	1.85277787257241	0.75682491083862
C	0.08137405552414	-1.47205109669417	1.42839876973421
C	1.27653084323950	-1.55816736214947	0.63067527344825
C	1.97073078185312	-0.18948991980000	0.70306002398300
C	-1.14206388311433	-1.70352532863422	0.56420182630172
C	-0.67616411559586	-1.86103943015993	-0.77221109683690
C	0.81792683169129	-1.72482317708837	-0.73212254069345
C	-1.16646754461073	-0.66557688313852	-1.55894227862253
C	-0.00339132153766	0.10958947896415	-2.00961187528278
C	1.21191957459663	-0.53962048237672	-1.50829876928700
C	1.90397907706939	0.39885277345621	-0.61695174911227
C	1.14600780434399	1.69587937760928	-0.57913792263264
C	-0.07798812314603	1.46386717574472	-1.44304413235881
H	0.12408285497322	0.31875562315726	-0.02309767058691
H	-0.12506394156000	-0.32585362064762	0.00536152913082

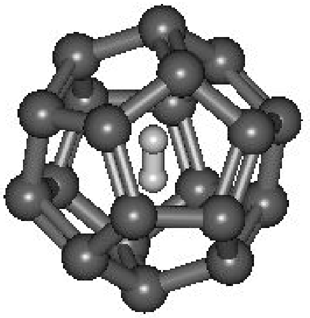

Figure S15. Optimized geometry of D_2 inside fullerene C_{20}.

16. xyz coordinates of initial geometry of 20 deuterons in a Pd_{44} cluster

Pd 1.388178 0.007538 1.320897
Pd 4.038535 0.021563 3.841405
Pd 0.106656 -2.131024 2.949187
Pd 1.394233 -0.020963 4.035220
Pd -1.320632 -0.034815 1.388081
Pd -1.388175 -0.007541 -1.320904
Pd 1.320633 0.034819 -1.388081
Pd 0.029285 -1.916377 -0.020032
Pd -0.029287 1.916378 0.020032
Pd -3.840527 -0.101357 4.038257
Pd -4.038533 -0.021574 -3.841407

Pd 3.840530 0.101353 -4.038252
Pd 0.085454 -5.571848 -0.058497
Pd -0.085453 5.571849 0.058503
Pd -2.938817 -2.147086 0.052194
Pd -0.042519 -2.070825 -2.993283
Pd 3.003281 -2.054689 -0.096125
Pd 2.938812 2.147090 -0.052191
Pd 0.042514 2.070824 2.993283
Pd -3.003283 2.054690 0.096123
Pd -0.106661 2.131013 -2.949164
Pd -4.034804 -0.076040 1.393707
Pd -1.394228 0.020966 -4.035234
Pd 4.034803 0.076046 -1.393705
Pd -2.582636 -2.032285 2.726158
Pd 1.410708 -3.754367 -1.462389
Pd 2.582635 2.032288 -2.726161
Pd -1.410707 3.754367 1.462385
Pd -2.717062 -1.978006 -2.633837
Pd 1.480755 -3.782609 1.313247
Pd 2.717065 1.978005 2.633837
Pd -1.480756 3.782610 -1.313249
Pd 2.642789 -1.894350 -2.767517
Pd -1.294677 -3.826269 1.382907
Pd -2.642793 1.894352 2.767515
Pd 1.294677 3.826271 -1.382909
Pd 2.777024 -1.948674 2.592705
Pd -1.364787 -3.797951 -1.392686
Pd -2.777024 1.948670 -2.592709
Pd 1.364787 3.797951 1.392688
Pd -1.190448 -0.061099 4.099402
Pd 4.099268 0.050050 1.191171
Pd 1.190449 0.061103 -4.099402
Pd -4.099268 -0.050048 -1.191171
H -0.047654 -3.739354 0.068984
H -0.034680 3.745763 0.045121
H -2.649014 0.008977 0.133044
H 2.620681 -0.005089 -0.070460
H -0.090903 0.044018 -2.636041
H 0.058117 -0.040642 2.568899
H -2.627380 -0.013257 2.658287
H 2.672227 0.015648 2.366129
H -2.579160 -0.003733 -2.440456
H 2.518871 0.040869 -3.098215

H 1.113574 2.075627 -1.354229
H 1.411615 2.013027 1.384500
H -1.433872 1.922664 1.454062
H -1.483402 -2.026138 -1.478570
H -1.442393 1.919558 -1.239826
H -1.220732 -2.029550 1.410054
H 1.519756 -2.113360 -1.579122
H 1.482960 -2.017432 1.481010
H -0.008770 0.387453 0.006211
H 0.001353 -0.312424 -0.002153

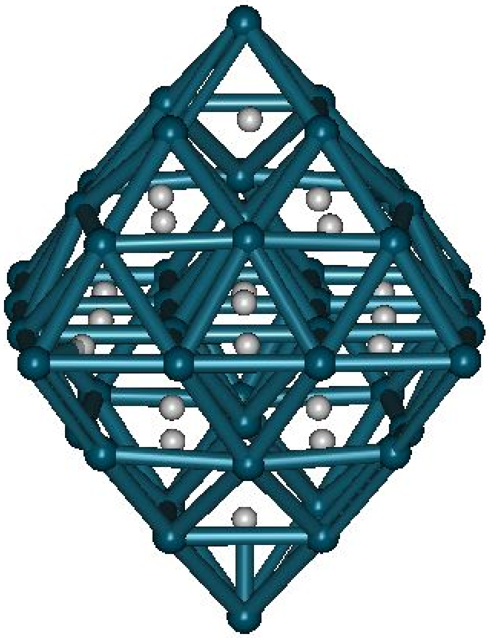

Figure S16. Initial geometry of 20 deuterons in a Pd_{44} cluster.

17. xyz coordinates of optimized geometry of 20 deuterons in a Pd_{44} cluster

Pd	1.47762791655697	0.02220443536216	1.35211696412195
Pd	4.32482658896383	0.20335145600947	3.90981625341421
Pd	0.17044038595399	-2.10415864885505	2.92269974238948
Pd	1.57068781481388	0.08910391084296	4.03077823686480
Pd	-1.27830394788025	0.01786806744875	1.38600071043244
Pd	-1.44506392441251	0.10439710632782	-1.38428196041719
Pd	1.34399639657010	0.06784362082868	-1.40771595907100
Pd	0.03609907061709	-1.97057607021771	-0.03513397489713
Pd	0.00461750833399	2.15848491495776	0.00027395495350
Pd	-3.94146749103389	0.14550048945597	4.07478683444799
Pd	-4.29873063136523	0.04714021423761	-4.02247407074234
Pd	4.02385153434850	0.25538165789668	-4.09876001052494

Pd	0.02785858357524	-5.77625873955044	-0.24948471521605
Pd	0.01583497063556	5.96374474196507	0.20281048759914
Pd	-2.83613135449369	-2.06488424547817	-0.02791355538803
Pd	-0.04698489522844	-2.04557692631253	-3.15763690936057
Pd	2.96143708435226	-2.05076717221106	-0.07262288178838
Pd	2.82017427348356	2.17566567403960	-0.01435100112839
Pd	0.12022796594325	2.14890384705943	2.87671675492597
Pd	-2.92264096270379	2.17598359939918	0.03911972284441
Pd	-0.17189665214714	2.25933516946076	-2.86663246845743
Pd	-3.99182206883650	0.03726798857486	1.40545904844152
Pd	-1.50963179806972	0.14100228331619	-4.15073438149086
Pd	4.06662293926881	0.14254553858207	-1.43074707316136
Pd	-2.63033430991389	-1.95262698010511	2.75488950811199
Pd	1.36070450096915	-3.82247507621837	-1.48473518758410
Pd	2.63940590837397	2.11193474075571	-2.69604233535629
Pd	-1.31838460685575	4.02296506182123	1.43734972499448
Pd	-2.86302091901835	-1.92989680608100	-2.81001792390438
Pd	1.52201090821287	-3.94578678408267	1.29117968248788
Pd	2.89505272556290	2.13612357320389	2.68840781279936
Pd	-1.48488433273807	4.14530751375120	-1.33730479265014
Pd	2.71356122624179	-1.85658313928027	-2.86212732353199
Pd	-1.30443158030117	-3.90238115049802	1.30505847719974
Pd	-2.57919770682449	2.03369201744763	2.80389606318329
Pd	1.32372731723168	4.12445660857426	-1.36150788428781
Pd	2.97036350734955	-1.98304696318406	2.67959526713206
Pd	-1.43426272629867	-3.90062932322450	-1.48243255893690
Pd	-2.92952983071291	2.21356800456608	-2.75312983489379
Pd	1.51845816477686	4.12249139881729	1.42424151710769
Pd	-1.17041351312793	0.06712892909061	4.13887711473521
Pd	4.16259646563814	0.07473367377024	1.27750799469819
Pd	1.25109114352302	0.17083838925645	-4.14751036179226
Pd	-4.18108648621839	0.07930742079425	-1.30372727902862
H	0.10083386796850	-5.11131462096656	1.33182855638824
H	-0.05298777707019	5.31345395032548	-1.42089308934465
H	-4.33243904684000	-1.08524587176180	0.04940475240101
H	4.43529097522914	-1.01874497787300	-0.09243446283845
H	-0.17408600845666	1.28828011675644	-4.36612399578904
H	0.23371977043397	-1.04168279514606	4.37618784743347
H	-2.66406259329305	-0.99054029514585	4.31199997876778
H	3.08696326891849	-0.96487026183658	4.17910378579560
H	-3.01402967663699	0.40686638563024	-2.99032385145367
H	2.71222029970703	-0.88334467389728	-4.39676312798066
H	1.30035493069712	3.27695889568812	-2.94444809149772

H	1.51160791396245	3.25018088584630	2.97521814019212
H	-2.90872569633014	3.22779614316013	1.49466639064746
H	-1.55976579886037	-3.07120516325404	-3.05353705499333
H	-3.06502153116492	3.31081780095540	-1.35462452847074
H	-1.33183915460158	-3.13971540657043	2.93422260016386
H	2.97036011382090	-3.09691661225414	-1.54677511862553
H	1.65987561136706	-3.16619604470196	2.88945603847854
H	0.09111878667847	0.41705244538383	0.15210045816020
H	-0.20125541864552	-1.20163092265336	-1.69559465670962

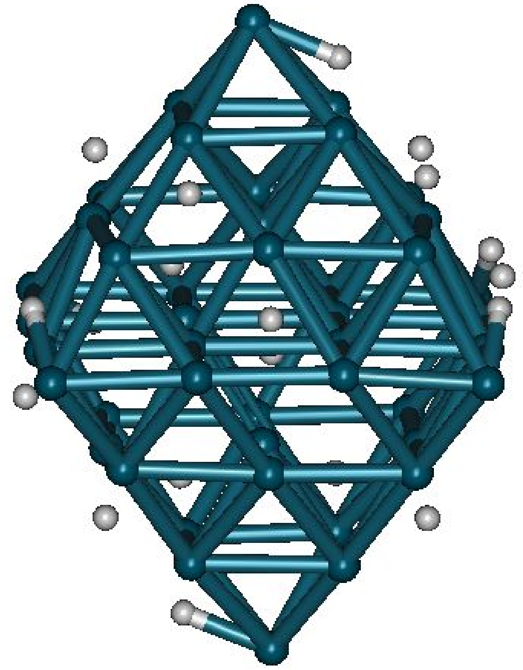

Figure S17. Optimized geometry of 20 deuterons in a Pd_{44} cluster.

18. The image depicts the structure immediately before the expulsion of two deuterium atoms from the SWCNT (2,2) nanotube.

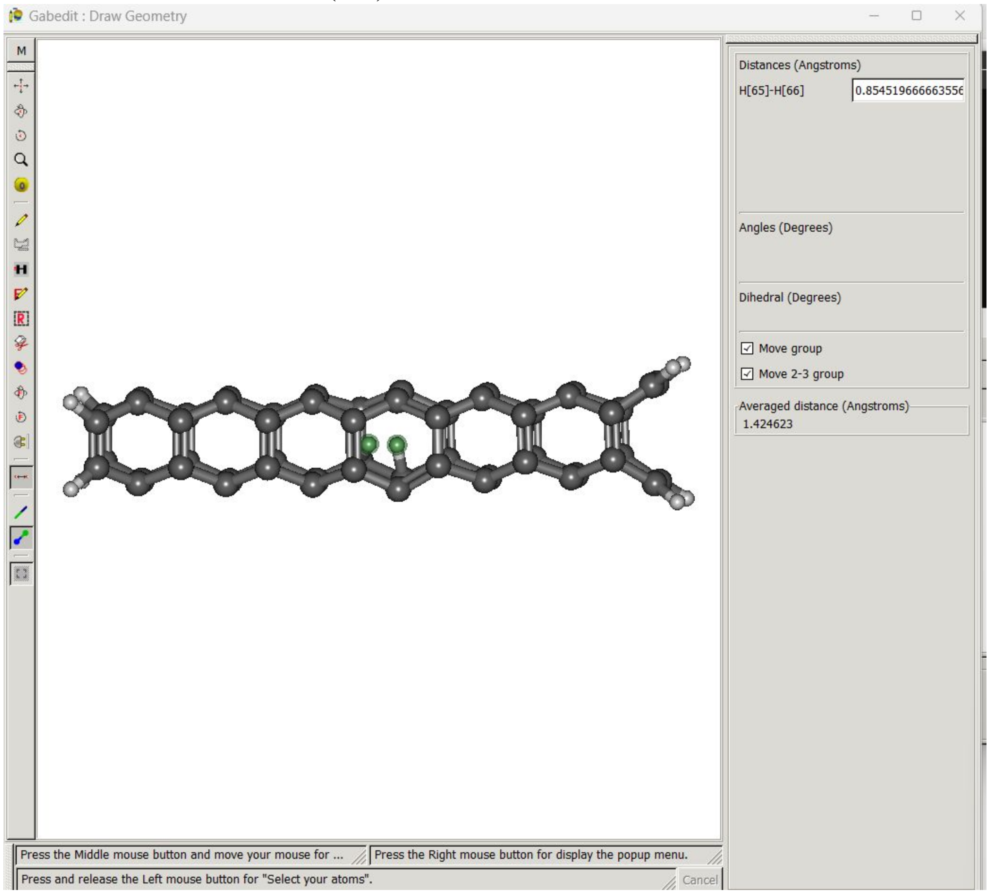

Figure S18. Optimized geometry of D_2 inside SWCNT (2,2) when the D–D distance became 0.85 Å in a relaxed surface scan

19. The image shows the structure with two deuterium atoms expelled from the SWCNT (2,2) nanotube.

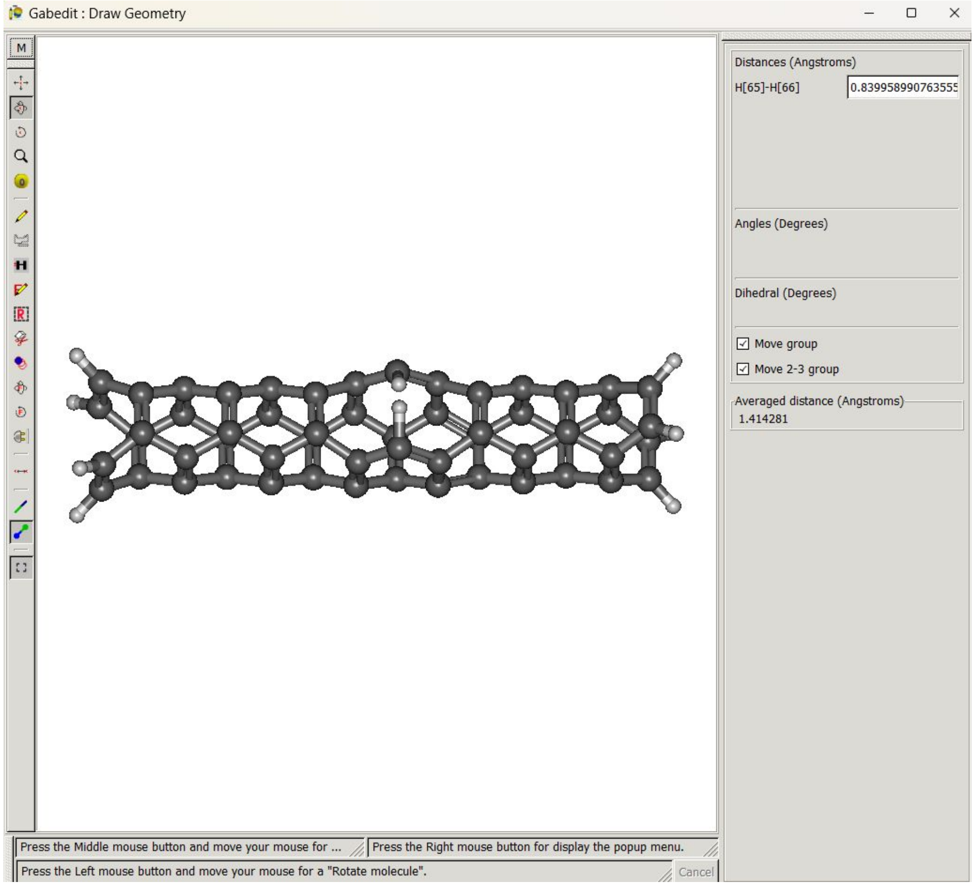

Figure S19. Optimized geometry of D_2 inside SWCNT (2,2) when the D–D distance became 0.84 Å in a relaxed surface scan

20. The image presents the plots of $V(r) - E$ against r of all chemical systems in this study, used for illustrating their Coulomb barriers of the D–D nuclear fusion.

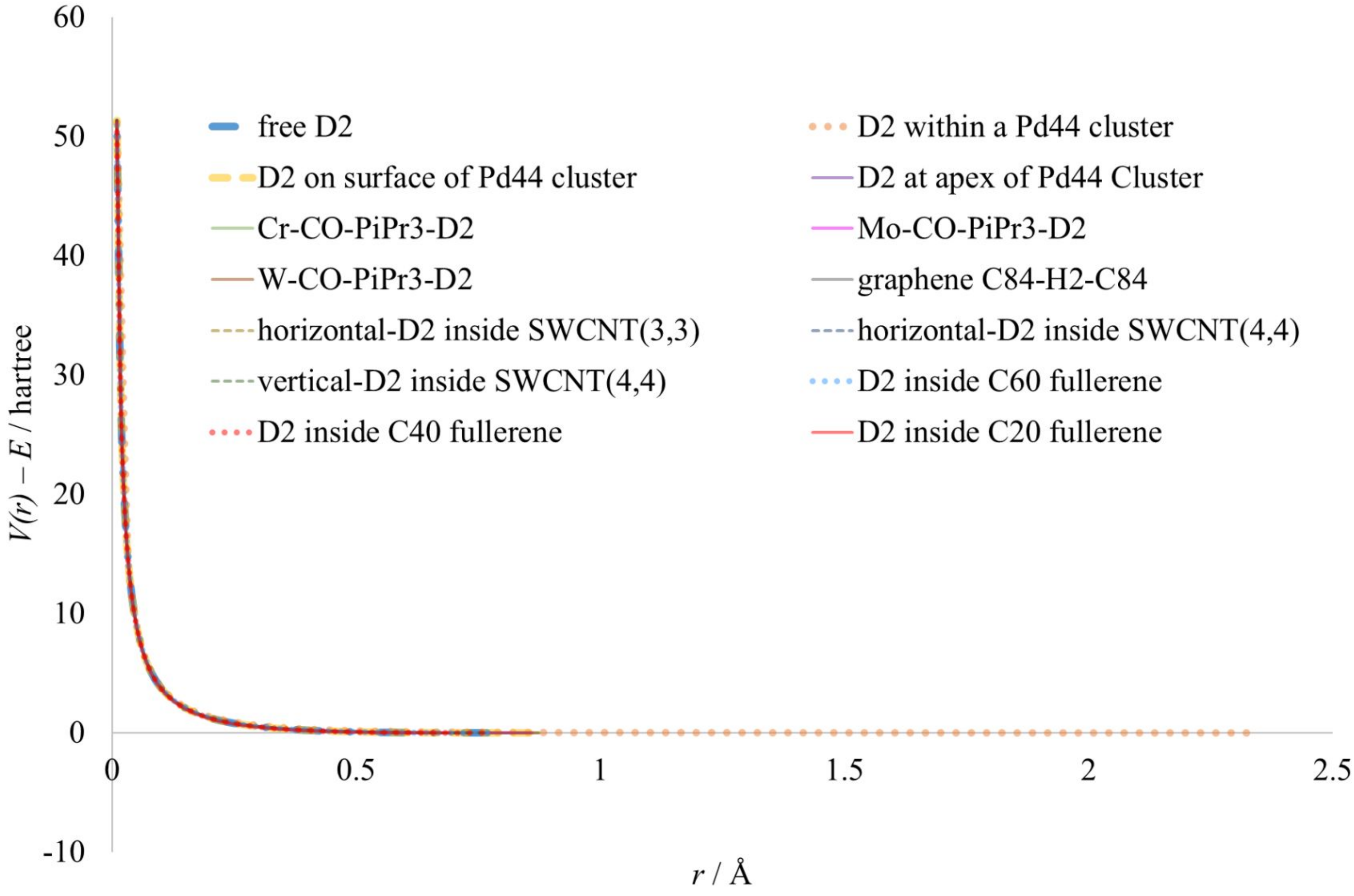

Figure S20. Plots of $V(r) - E$ versus r of all chemical systems in this study.
